# Deciphering environmental factors and defense response of rice genotypes against sheath blight disease

**DOI:** 10.1016/j.pmpp.2022.101916

**Published:** 2022-11

**Authors:** R. Naveenkumar, A. Anandan, Vineeta Singh, S.R. Prabhukarthikeyan, C. Parameswaran, G. Sangeetha, A. Mahender, U. Keerthana, P.K. Singh, B.C. Patra, Jauhar Ali

**Affiliations:** a Indian Council of Agricultural Research (ICAR)-National Rice Research Institute (NRRI), Cuttack, Odisha, 753006, India; b Institute of Agricultural Sciences, Banaras Hindu University (BHU), Varanasi, Uttar Pradesh, 221005, India; c Department of Agriculture, Karunya Institute of Technology and Sciences, Karunya Nagar, Coimbatore, Tamil Nadu, 641114, India; d ICAR-Indian Institute of Seed Science, Regional Station, Bangalore, 560065, Karnataka, India; e ICAR-Indian Institute of Horticultural Research (IIHR), Bangalore, 560089, Karnataka, India; f Rice Breeding Innovation Platform, International Rice Research Institute (IRRI), Los Banos, Laguna, 4031, Philippines

**Keywords:** Rice germplasm, *Rhizoctonia solani*, Disease score, Antioxidants, Enzymatic activity, Pearson correlation, Principal component analysis

## Abstract

Sheath blight (ShB) is one of the most serious diseases in rice, leading to severe yield losses globally. In our study, we evaluated a total of 63 rice genotypes for resistance against sheath blight disease by artificial inoculation over two seasons under field conditions and studied the weather parameters associated with disease incidence. Based on two years of testing, 23 genotypes were found moderately resistant, 38 were moderately susceptible, and 2 exhibited a susceptible reaction to sheath blight disease. Among the specific four genotypes (IC283139, IC283041, IC283038, and IC283023) of the moderately resistant group exhibited less disease reaction in comparison with check variety Tetep. Further, the correlation of percent disease index (PDI) with weather parameters revealed negative associations between PDI and maximum temperature, minimum temperature, low rainfall and the positive association with maximum relative humidity (RH) suggest that very low temperature or high precipitation might have a negative impact on pathogen establishment. In addition, the sheath blight-linked SSRs were assessed using distance and model-based approaches, results of both the models revealed that genotypes distinguished the resistant population from the susceptible one. From the output of two years of principal component analysis, two genotypes from each group of moderately resistant, moderately susceptible and susceptible were studied for their biochemical reaction against the sheath blight pathogen. The biochemical study revealed that the accumulation of defense and antioxidant enzymes, namely, polyphenol oxidase, peroxidase, total phenol, phenylalanine ammonia-lyase, catalase, and superoxide dismutase, were higher in moderately resistant genotypes, but was observed to be lower in moderately susceptible and susceptible genotypes. The statistical analysis revealed the enzyme activities (defense and antioxidant) exhibited a strong negative correlation with area under the disease progress curve (AUDPC) and influence of weather parameter RH. This demonstrates that the environment factor RH plays a major role in imparting the resistance mechanism by decreasing the enzymes activities and increasing PDI. This study found that the identified novel resistant genotype (IC283139) with purple stem base demonstrated improved resistance against sheath blight infection through a defense response and the use of antioxidant machinery.

## Highlights

• First report on novel genotype (IC283139) is highly moderately resistant to sheath blight disease under field conditions.
• Markers utilized for the study was revealed the resistant and susceptible germplasm in separate groups.
• The resistant genotype exhibited antioxidant machinery as well as defense response resistance to sheath blight infection.
• Weather parameters like maximum relative humidity and temperature played a critical role in sheath blight disease development.

## Graphical abstract

**Figure:**
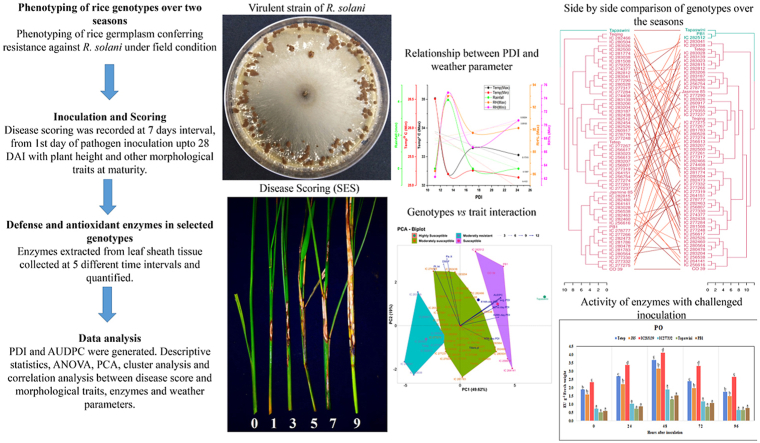

## 1 Introduction

Rice (*Oryza sativa* L.) is a vital staple food for 50% of the world's population and is especially important for the rapidly increasing population of South Asian countries. China and India are ranked top among the major rice-producing countries globally [1]. Rice cultivation in India covers about 43 million hectares with a production of 115 million tons of milled rice and a productivity of 2.7 tons ha^−1^ [2]. However, rice productivity is affected by several pathogens, which often plays a major constraints on cultivation. Diseases pose a serious challenge to rice cultivation and greatly diminish yield. Even though rice is affected by fungi, bacteria, and viruses, the fungal diseases sheath blight (caused by *Rhizoctonia solani*), blast (caused by *Magnaporthe oryzae*), and brown spot (caused by *Bipolaris oryzae*) are prominent destructive pathogens [3]. Among them, *Rhizoctonia solani* Kuhn causing sheath blight of rice (teleomorph: *Thanatephorus cucumeris* (Frank) (Donk)) is a widespread destructive pathogen, considered as the second most significant disease after rice blast [4]. Management of *R. solani* is a tough challenge due to the persistence of sclerotia in adverse environmental conditions and wide host range, which includes important field and horticultural crops, namely, wheat, potato, carrot, tomato, soybean, maize, and cotton [5].

So far, resistance breeding for *R. solani* has been unsuccessful in rice because of the inability to identify a stable resistance source [4] across locations and the favorable role of weather parameters in establishing sheath blight disease. On the other hand, the excessive use of fungicides increases health risks, is toxic to other eukaryotic organisms, and poses severe environmental hazards [6]. Thus, developing rice cultivars with resistance to sheath blight disease would be an environmentally friendly strategy for managing sheath blight disease [7]. The quantitative nature of resistance could be compatible with durable or horizontal resistance [1].

To overcome this disease incidence, there is a need to evaluate rice cultivars to identify broad-spectrum resistance genes and their defense and antioxidant enzyme interactions with diverse rice cultivars [8]. Hence, it is necessary to understand the defense- and antioxidant-mediated disease resistance mechanisms involved in *R. solani* and rice interactions. Various researchers reported the activity of polyphenol oxidase (PPO), peroxidase (PO), phenylalanine ammonia-lyase (PAL), catalase (CAT), and superoxide dismutase (SOD) in defense and oxidative responses against *R. solani* through diverse studies [9]. Plants have a well-developed antioxidant defense system that responds to both abiotic and biotic stresses and mitigates the adverse effects of reactive oxygen species (ROS) [10]. These ROS functions may be directly involved in the development of defense mechanisms or indirectly through synergistic interactions with some other signaling molecules [11]. To minimize the toxic effects of ROS oxidative damage, plants activate ROS scavengers such as CAT and SOD to regulate the cytotoxic effects of free radicals [12]. Peroxidase and PPO catalyze the oxidation of phenolic compounds through a PPO-PO-H_2_O_2_ system [13]. PAL is the crucial enzyme that triggers salicylic acid production, which activates systemic resistance in plants, and it provides a precursor of the biosynthesis of lignin and some other phenolics that accumulate in response to pathogen infection [10]. Several studies have reported the responses of defense mechanisms and ROS scavengers against stresses. However, the association between weather parameters and enzyme (defense and oxidative) activity related to sheath blight has not been reported previously. Thus, by keeping this in view, our study was formulated with the following objectives: to understand (i) the genetic diversity and identification of stable sources of rice genotypes with resistance against *R. solani, (*ii) the role of weather parameters in the progression of sheath blight disease, and (iii) the biochemical-based defense mechanism and its association with weather parameters.

## 2 Materials and methods

### 2.1 Experiment 1

#### 2.1.1 Plant material and pathogen

A total of 63 rice genotypes were collected from the ICAR-National Rice Research Institute (NRRI), Cuttack, Odisha, India, and Banaras Hindu University (BHU), Varanasi, Uttar Pradesh, India were used for the present study (Table S1). The experiment was conducted at the experimental farm of BHU (28.18^o^ N, 38.03^o^ E, and 75.5 masl) during the wet season (kharif) 2017 (season I) and 2018 (season II) to evaluate the rice germplasm against *R. solani*. For this experiment, the genotypes were screened using a highly virulent AG1 IA isolate of *R. solani* (MTCC-12227) obtained from the Department of Mycology and Plant Pathology, Institute of Agricultural Sciences, BHU.

#### 2.1.2 Screening of rice genotypes against *R. solani*

Seedlings were raised in an elevated nursery bed and irrigated at regular intervals to maintain enough moisture for their better growth. They were carefully uprooted from the nursery bed after 30 days and transplanted into the main field at two seedlings per hill with 20 × 15 cm spacing. The main field experiment followed a randomized block design with a plot size of 12 m^2^ and susceptible checks Pusa Basmati-1 [6] and Tapaswini [14] and resistant checks Tetep [6], Jasmine 85, and Teqing [5] planted in each block with three replications for each treatment. A basal application of 50% nitrogen (N), 100% phosphorus (P), and (100%) potash (K) was applied (120:60:60 kg NPK ha^−1^) during transplanting, and the rest (50% N) was applied upon the second-hand weeding. The field was maintained weed-free by manual weeding on the 25th day and the 45th day after transplanting. At the booting stage of the crop, the plants were inoculated with immature sclerotia from 3- to 4-day-old highly virulent strains of *R. solani* [6]*.* The morphological traits plant height (cm), tiller number per plant, panicle length (cm), and days to 50% flowering were measured, and disease scoring was calculated as described below.

#### 2.1.3 Disease assessment

Disease scoring was carried out using a 0–9 scale of the International Rice Research Institute's standard evaluation system (SES) [15], and disease severity was recorded at 7-day intervals after pathogen inoculation up to 28 days after inoculation [16]. Relative lesion height (RLH) was calculated using the formula described by Sharma and Teng [17]:

$$\text{RLH}=\frac{\text{Maximum height at which lesion appears}}{\text{Plant height}}\text{ x }100$$

Per cent disease index (PDI) was calculated using the formula given by Baker and Wheeler [18]:

$$\text{PDI}=\frac{\text{Sum of all ratings}}{\text{Total no}.\text{ of observations x Maximum rating scale}}\text{ x }100$$

The area under the disease progress curve (AUDPC) was calculated as follows [10,19]:

$$\text{AUDPC}=\overset{n}{\underset{i=1}{\sum }}\left\{\left[\left(\text{Xi}+1+\text{ Xi}\right)\right]/2\text{ x }\left(\text{ti}+1–\text{ ti}\right)\right\}$$

where X_i_ is the disease index expressed as a proportion at the ith observation, t_i_ is the time (days after planting) at the ith observation, and n is the total number of observations.

#### 2.1.4 DNA isolation and quantification

Young leaf samples were collected from 21-day-old seedlings of 63 genotypes and the total genomic DNA of all the genotypes was isolated using an automated tissue homogenizer (1600 Mini G®, NJ) in CTAB as described by Murray and Thomson [20]. DNA concentration was estimated by using 0.8% agarose gel electrophoresis. The sample DNA was diluted to 50 ng/μL and amplified using 17 sheath blight-linked simple sequence repeats (SSRs) documented from earlier reports of bi-parental mapping that spread across the rice chromosomes (Table S2).

#### 2.1.5 PCR amplification and visualization of markers linked to sheath blight resistance

PCR amplification was carried out in a thermocycler (Bio-Rad) using 10 μL of PCR mixture containing 15 ng of genomic DNA, 1x PCR buffer, 200 μM of dNTP mix, 4 pmol of each forward and reverse primer, 2 mM of MgCl_2_, and 1U of Taq DNA polymerase. In the thermocycler, initially, the reaction mixture was denatured for 5 min at 94 °C, followed by 35 cycles of denaturation for 1 min at 94 °C, annealing for 1 min at 55 °C, extension for 2 min at 72 °C, and then final extension for 10 min at 72 °C. Aliquots of 8 μL of the PCR product were electrophoresed in 3% agarose gel that had 0.8 μg/mL of ethidium bromide in 1XTBE (pH 8.0), and the size of the amplicons was determined by using a 50-bp DNA ladder. The gel was run at 100 volts (2.5 V/cm) for 3 h and the amplified products were visualized and photographed under a UV light source in a gel documentation system (Bio-Rad) (Fig. S1).

#### 2.1.6 Genetic diversity parameters, genetic relatedness, and population assignment test

Seventeen sheath blight-linked SSRs were used to assess the genetic diversity among the 63 genotypes. Allele size of marker data was generated for each genotype based on the presence or absence of bands and arranged in the form of a binary data matrix as discrete variables. Allele number, major allele frequency, gene diversity, heterozygosity, and the polymorphic information content (PIC) for each SSR locus were calculated by using Power Marker software version 3.25 [21]. Further, the allelic data were subjected to Nei's coefficient for dissimilarity index with a bootstrap value of 1000 and the unrooted unweighted neighbor-joining tree was constructed using Darwin 6.0 software [22]. A population assignment test was performed using the GenAIEx 6.5.0 cross-platform package [23]. The genotypic data for the genotypes under study were analyzed for possible population structure with the model-based program Structure 2.3.4 [24] using a length of the burn-in period of 1,00,000, followed by 100,000 Markov chain Monte Carlo (MCMC) replications. At least ten runs of Structure were carried out by setting the number of sub-populations (K) from K = 1 to K = 10. To find the true K-value, ad hoc statistics ΔK were followed [25] using Structure Harvester version 0.6.94 [26].

#### 2.1.7 Weather data

Weather data from the experimental farm were obtained from the agro-meteorological observatory, BHU, Varanasi, located at 28°18′ N latitude, 38°03′E longitude, and altitude of 75.5 masl. Parameters such as temperature (maximum and minimum), sunshine hours, rainfall, and relative humidity (maximum and minimum) were collected from the 36th to 40th standard week and used in establishing the effect of weather parameters on disease incidence.

### 2.2 Experiment 2

#### 2.2.1 Enzyme extraction for defense and related antioxidant enzymes

Based on the genotypic and phenotypic information from two seasons, genotypes exhibiting higher resistance with minimum disease incidence than the positive check (Tetep) were categorized as moderately resistant (IC283139), moderately susceptible genotypes (Jasmine 85 and IC 277332), and highly susceptible genotypes (Pusa Basmati-1 and Tapaswini) and were selected to understand the biochemical-based defense mechanism-mediated resistance against *R. solani* in rice. The experiment was carried out under net house conditions at ICAR-NRRI during the wet season of 2019. Uniform-sized seeds of all the genotypes were selected to break their dormancy by placing them in a hot-air oven at 50 °C for 45 h. The seeds were surface-sterilized to minimize contamination with 2.5% sodium hypochlorite for 20 min and washed five times to remove any traces of disinfecting agents using sterile distilled water. The sterilized seeds were pre-germinated in a Petri dish on a paper towel and moistened with distilled water for 72 h at 28 °C in an incubator. After incubation, well-germinated seeds were transferred to pots containing the sterilized potting mixture of soil:sand:farmyard manure (FYM) at 3:1:1 (w/w/w). Twenty-five days later, the seedlings were transplanted into an earthen pot by maintaining three plants per pot. Three separate pots were maintained as three biological replicates in a completely randomized manner with the necessary dose of fertilizer and moisture.

Twenty days after transplanting (45-day-old plants), the plants were inoculated with strain AG1 IA (a highly virulent strain of *R. solani*) as described by Goswami et al. [6]. The plants without pathogen inoculation served as controls. Rice leaf sheaths were collected at different time intervals such as 0 h, 24 h, 48 h, 72 h, and 96 h after pathogen inoculation. Three plants were sampled from each replication and the collected samples were transferred immediately into liquid nitrogen by wrapping them in aluminium foil. The collected samples were stored at −80 °C until used for enzyme extraction. Extraction of enzymes was carried out by homogenizing 1 g of rice leaf sheath tissue using a mortar and pestle pre-chilled (4 °C) with 2 mL of 0.1 M sodium phosphate buffer at 4 °C with pH 7.0, and the grounded sample was centrifuged at 10,000 rpm for 20 min at 4 °C. The upper aqueous solution was collected and served as a crude enzyme. The activity of polyphenol oxidase (EC 1.10.3.1), peroxidase (EC 1.11.1.7), phenylalanine ammonia-lyase (EC 4.3.1.24), and total phenol was estimated as described by Mayer et al. [6], Hammerschmidt et al. [27], Dickerson et al. [28], and Zieslin and Ben-Zaken [29], respectively. The enzyme extracted using 0.2 M of citrate phosphate buffer with pH 6.5 was used to determine superoxide dismutase (EC 1.15.1.1) [30] and the enzyme extracted with potassium phosphate buffer (100 mM, pH 7.5) was used for the estimation of catalase (EC 1.11.1.6) as described by Chaparro-Giraldo et al. [31]. These defense and antioxidant enzymes unit (EU) was expressed as per gram fresh weight basis.

#### 2.2.2 Weather data

Weather parameters such as temperature (maximum and minimum), sunshine hours, rainfall, and relative humidity (maximum and minimum) of the experimental site, NRRI (20°27′09″ N, 85°55′57″ E, 26 masl), Cuttack, were obtained from the NRRI agro-meteorological observatory for the experimental period to establish the effect of weather parameters on the activity of defense and antioxidant enzymes.

### 2.3 Statistical analysis

Descriptive statistics, analysis of variance (ANOVA), and principal component analysis (PCA) were used with 63 rice genotypes for 10 traits to identify sheath blight-resistant genotypes based on their performance over the season. Descriptive statistics and ANOVA used Windostat 7.5 software. PCA and biplot analysis were conducted using the PCA function from the *FactoMineR* package [32] in R version 3.6.3. Agglomerative hierarchical clustering with the complete linkage method was adapted to compare genotypes of two seasons side by side using the function *tanglegram* [33] in R. To understand the interaction between the morphological traits and disease score and the role of weather parameters on disease progression, Pearson correlation was carried out using the *corrplot* functions from the *corrplot* package [34] in R.

Among the 63 genotypes, six representative genotypes were selected based on their resistance and susceptibility reaction to understand the induced systemic resistance (ISR) reaction against sheath blight. ANOVA for six enzymes over different time points of six genotypes and their interaction and a bar graph depicting the enzyme activity of six genotypes across four times were displayed using Windostat 7.5 and Microsoft Excel (2016), respectively.

## 3 Results

### 3.1 Phenotypic and genotypic heterogeneity of genotypes and association between variables

#### 3.1.1 Distribution and grouping of diverse rice genotypes

Descriptive statistics of the population panel suggest that the population has a wide range of variations over the seasons (2017 and 2018). The mean, median, and mode were similar and the distribution frequency was normal for most of the traits other than plant height, AUDPC, and PDI on the 28th day from inoculation. The coefficient of variation was considerable and it differed from 8.72% (7th day PDI) to 55.97% (21st day PDI) for the wet season of 2017 (Table 1), whereas, in the wet season of 2018, it ranged from 6.90% (days to 50% flowering) to 44.72% (14th day PDI) (Table 2). The skewness value of all the traits were normal over the seasons, while the PDI of the 14th day was >3 in the wet season of 2017 (Table 1). Similarly, kurtosis values of the wet season of 2017 were >3 for most of the traits, while panicle length, plant height, number of tillers, and days to heading were less than 1. In the wet season of 2018, all the traits were normal except PDI on the 7th day which exhibited kurtosis of >3. Hence, the distribution of the whole panel was platykurtic, which suggests that the genotypes were not just concentrated near the mode but rather distributed uniformly in between the extreme range values. ANOVA revealed significant (*P > 0.01*) variation among genotypes and seasons for all the traits observed except for the 7th day PDI at the genotypic level. The interactions between season and genotypes for panicle length, number of tillers, 7th day PDI, and AUDPC were non-significant (Table 3).

**Table 1:** Descriptive statistics of different traits of wet season 2017.

Characters	Mean	Standard deviation	Lower 95% CI of mean	Upper 95% CI of mean	Variance	Skewness	Kurtosis	Coefficient of variation	Mode	Minimum	Median	Maximum
Plant height	107.91	27.18	101.07	114.76	738.74	0.20	−0.97	0.25	67.80	66.23	108.30	163.50
Panicle length	23.32	3.24	22.50	24.13	10.49	0.12	−0.57	0.14	23.67	17.02	23.57	31.16
Tillers/plant	10.64	3.84	9.67	11.60	14.78	0.49	−0.36	0.36	8.30	4.10	10.10	20.80
Days to 50% flowering	100.98	12.32	97.88	104.09	151.69	0.78	0.64	0.12	95.00	79.00	100.00	138.00
Average PDI	16.41	6.41	14.79	18.02	41.15	2.58	11.05	0.39	11.11	10.00	15.56	50.00
AUDPC	480.76	192.07	432.39	529.13	36889.47	2.96	12.81	0.40	350.00	318.89	412.22	1516.67
7th day PDI	11.18	0.98	10.94	11.43	0.95	1.37	19.40	0.09	11.11	6.67	11.11	15.56
14th day PDI	13.20	6.46	11.58	14.83	41.69	5.07	30.63	0.49	11.11	11.11	11.11	55.56
21st day PDI	17.07	9.55	14.66	19.47	91.25	1.80	3.31	0.56	11.11	11.11	11.11	55.56
28th day PDI	24.18	12.07	21.15	27.22	145.66	1.31	4.65	0.50	33.33	11.11	28.89	77.78

PDI-Percent disease index, AUDPC-area under the disease progress curve.

**Table 2:** Descriptive statistics of different traits of wet season 2018.

Characters	Mean	Standard deviation	Lower 95% CI of mean	Upper 95% CI of mean	Variance	Skewness	Kurtosis	Coefficient of variation	Mode	Minimum	Median	Maximum
Plant height	94.19	18.34	89.57	98.81	336.37	0.50	1.36	0.19	NA	56.54	94.94	157.02
Panicle length	21.57	3.21	20.76	22.38	10.33	0.38	−0.56	0.15	19.91	15.72	20.61	28.72
Tillers/plant	8.91	3.05	8.14	9.68	9.32	0.94	0.85	0.34	7.70	4.30	8.20	18.60
Days to 50% flowering	103.38	7.14	101.58	105.18	50.95	1.22	1.40	0.07	102.00	92.00	102.00	126.00
Average PDI	27.79	6.23	26.22	29.36	38.84	0.47	2.41	0.22	25.00	11.11	28.06	49.07
AUDPC	798.13	189.71	750.35	845.90	35988.49	0.92	2.53	0.24	700.00	350.00	775.83	1464.81
7th day PDI	12.28	2.37	11.68	12.88	5.62	2.17	4.14	0.19	11.11	11.11	11.11	21.11
14th day PDI	16.30	7.29	14.47	18.14	53.17	1.65	2.84	0.45	11.11	11.11	13.33	44.44
21st day PDI	35.99	9.30	33.64	38.33	86.56	0.22	1.05	0.26	33.33	11.11	33.33	59.26
28th day PDI	46.59	11.05	43.81	49.37	122.16	−0.62	1.97	0.24	44.44	11.11	44.44	77.77

PDI-Percent disease index, AUDPC-area under the disease progress curve, NA - not available (the data of plant height does not have any repeating number).

**Table 3:** Analysis of variance (ANOVA) for various traits of rice genotypes over seasons.

Source of variation	DF	Mean sum of squares									
		Plant height	Panicle length	Tillers/Plant	Days to 50% flowering	7th day PDI	14th day PDI	21st day PDI	28th day PDI	Average PDI	AUDPC
Seasons (S)	1	11866.29**	192.44**	187.12**	361.92	76.13**	605.84**	22558.14**	31627.29**	4426.57**	6345441.36**
Genotypes (G)	62	1503.03**	34.52**	41.62**	295.72**	6.72^ns^	128.82**	228.22**	294.76**	378.04**	64981.03**
S x G	62	647.18**	7.11^ns^	6.5^ns^	109.57**	6.42^ns^	60.87*	127.35**	240.84**	313.28**	32910.36^ns^
Error	126	315.69	10.06	8.83	0.00	6.27	41.45	32.43	59.86	151.33	36083.90

** -significance value at 0.01%, ns-non-significance.

Multivariate PCA was used to identify traits that affect sheath blight resistance and grouping of rice genotypes based on similarity in expression of traits under a given environment. The genotype by trait biplot of the wet season of 2017 presented in Fig. S2 captured 71% of the variation among the genotypes for the first two components. Component 1 explained 53.55% of the variation while component 2 explained 17.5% of the variation towards the total variability. Among the different traits studied, days to heading shared maximum variability, followed by panicle length and plant height, while AUDPC and PDI were grouped in an opposite component in the negative quadrant. Furthermore, the right side of the plot represented resistance and most of the moderately resistant (MR) genotypes, whereas the left side clustered moderately susceptible (MS) and susceptible genotypes. In the wet season of 2018, the first two components together explained 65.49% (PC1, 36.29%, and PC2, 29.25%) of the total variation (Fig. S3). A similar trend of trait and genotype grouping was observed in the wet season of 2018 as in the wet season of 2017. The biplot graph has separated the plots into two halves. Resistant genotypes were on one side (right) of the plot and susceptible genotypes were grouped on the opposite side (left) of the plot.

The PCA of pooled data over the seasons exhibited a similar trait and genotype grouping pattern of the wet season of 2017 and 2018. The first PC explained 49.62% of the variation and the second PC explained 19.0% of the variation, together accounting for 68.62% of the total variation. Among the traits studied, disease-related traits such as AUDPC, PDI (7–28 days), and average PDI were highly associated with PC1, while the morphological traits, namely, panicle length, plant height, and days to 50% flowering, had a constructive correlation with PC2 (Fig. 1). The traits plant height, AUDPC, average PDI, and 21st and 28th day PDI accounted for a maximum contribution towards the diversity, while tiller number per plant and 7th and 14th day PDI exhibited a minimum contribution (Fig. 1). Of the four quadrants, quadrants 1 (top left) and 4 (bottom left) accommodated moderately resistant (9) and moderately susceptible (48) genotypes in response to sheath blight resistance under field conditions.

**Fig. 1:**
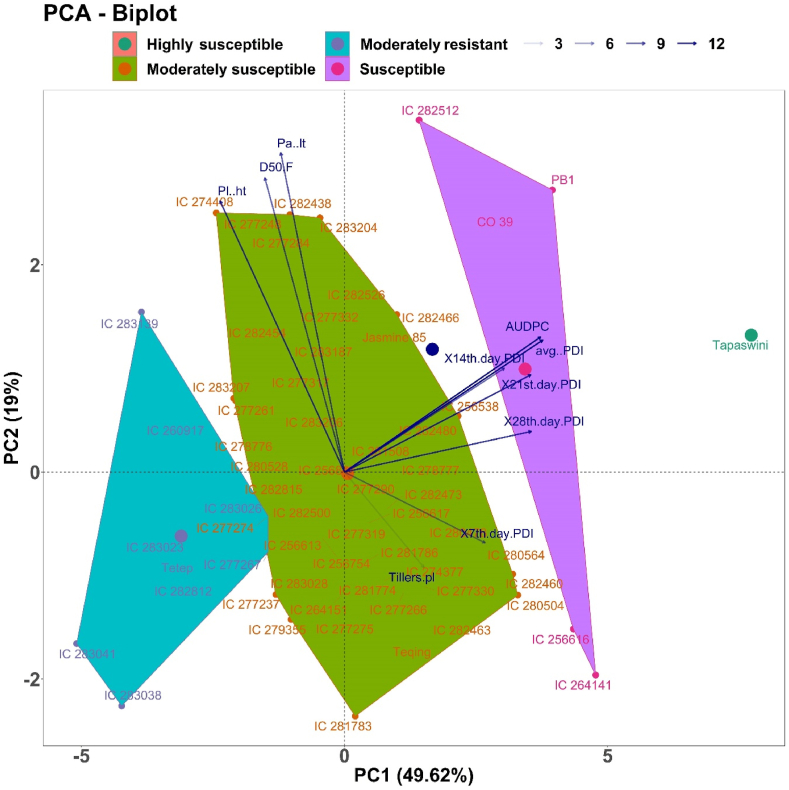
Biplot graph represents genotypes in two main principal components for traits associated with sheath blight disease over the season. The two PC axes together explained 68.62% of the total variation. The transparency of the vector indicates the contribution to the variation in the dataset, ranging from 3% (lightest) to 12% (darkest). The direction and length of the vector represent trait contribution to the first two components of the PCA. Genotypes are grouped into four based on their level of tolerance. Group 1 (elf Green with orange) is highly susceptible, Group 2 (light violet with pink) is susceptible, Group 3 (dark green with orange) is moderately susceptible, and Group 4 (cyan with purple) is moderately resistant. Note: Pl. ht: plant height, D50F: days to 50% flowering, Pa. lt: panicle length, AUDPC: area under the disease progress curve, avg. PDI: average percent disease index, X7th PDI: PDI of 7th day, X14th PDI: PDI of 14th day, X21st PDI: PDI of 21st day, X28th PDI: PDI of 28th day, Tillers Pl: number of tillers per plant.

In contrast, quadrants 2 and 3 together accounted for the members of moderately (5) and highly susceptible (1) genotypes that were less tolerant than those of quadrants 1 and 4. Further, the side-by-side hierarchical clustering of two seasons (Fig. S4) revealed a genotype grouping patterning according to their resistance reaction against the disease and confirmed PCA results in the wet season of 2017 and 2018. Two genotypes similar in their tolerance or susceptibility score were selected from the above four groups to understand the biochemical-based defense mechanism-mediated resistance against the pathogen *R. solani*.

#### 3.1.2 Genetic diversity

The panel population of 63 genotypes was genotyped using 17 SSRs representing each arm of the chromosome to understand the genetic relatedness. A total of 54 alleles were obtained with an average of 3.176 alleles per locus from the 17 loci (Table 4). The allele number differed from 2 (RM306, RM5529, RM335, RM178, RM5428, and RM101) to 5 (RM1350, RM334) per loci. The major allele frequency values varied from 0.351 (RM334) to 0.929 (RM81), with a mean of 0.612 per locus. Further, the highest PIC (polymorphic information content) value (0.684) and gene diversity (0.732) were observed for the primer RM334 and the lowest values (0.132 and 0.136 for PIC and gene diversity) were observed for the primer RM81. The average values of 0.427 and 0.491 were observed for all the markers studied per locus. In addition, the inbreeding coefficient values ranged from 0.071 to 1.000, with a mean of 0.954 per locus.

**Table 4:** Details of microsatellite markers used for genotyping the panel population and their estimated molecular genetic diversity parameters.

S. no.	Marker	Chromosome no.	Allele no.	Major allele frequency	Gene diversity	PIC	Inbreeding coefficient
1	RM306	1	2	0.552	0.495	0.372	1.000
2	RM5529	2	2	0.820	0.295	0.252	1.000
3	RM1350	3	5	0.606	0.580	0.540	1.000
4	RM16	3	3	0.705	0.429	0.354	1.000
5	RM3117	3	3	0.435	0.628	0.549	1.000
6	RM570	3	4	0.409	0.670	0.608	1.000
7	RM81	3	4	0.929	0.136	0.132	0.071
8	RM335	4	2	0.688	0.430	0.337	1.000
9	RM518	4	3	0.477	0.592	0.505	0.699
10	RM13	5	4	0.583	0.574	0.513	0.944
11	RM178	5	2	0.593	0.483	0.366	1.000
12	RM334	5	5	0.351	0.732	0.684	1.000
13	RM5428	8	2	0.649	0.456	0.352	1.000
14	RM3452	8	4	0.397	0.689	0.631	1.000
15	RM257	9	4	0.525	0.628	0.569	0.948
16	RM3823	9	3	0.774	0.370	0.334	1.000
17	RM101	12	2	0.906	0.170	0.155	0.880
	**Mean**		**3.176**	**0.612**	**0.491**	**0.427**	**0.957**

#### 3.1.3 Genetic relatedness by cluster analysis and population structure analysis

Cluster analysis was performed to find out the genetic distance and dissimilarity matrix using the unweighted neighbor-joining method. In the unrooted tree, rice germplasm was grouped into three major clusters (Fig. 2). Cluster 1 accounted for 18 accessions and was further divided into clusters 1a, 1b, and 1c with 13, 2, and 5 genotypes, respectively. Similarly, cluster 2 accommodated 40 genotypes and was further subdivided into clusters 2a and 2b with 23 and 17 genotypes, respectively. In addition, cluster 3 covered only five genotypes, which was further sub-grouped into cluster 3a with three genotypes and cluster 3b with two genotypes.

**Fig. 2:**
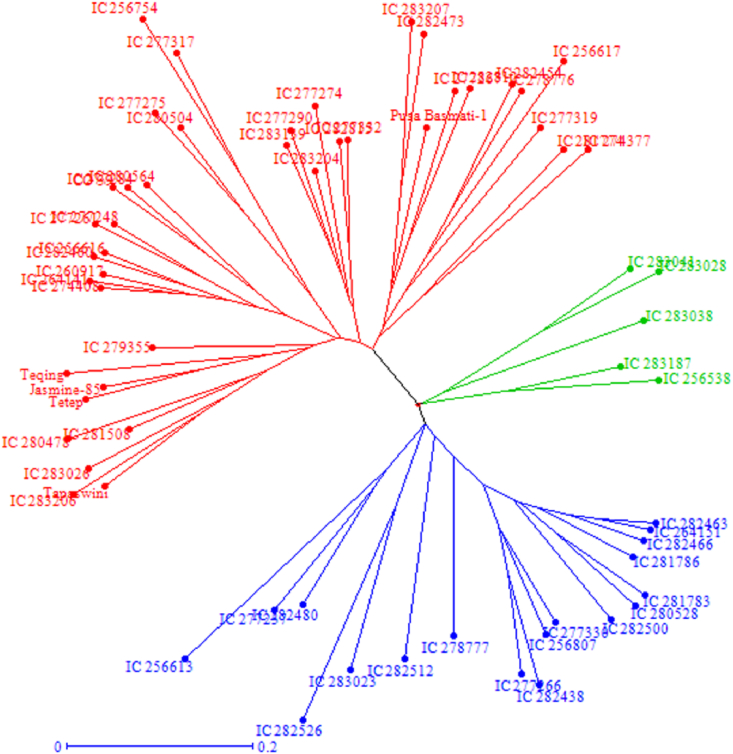
Unrooted tree using the unweighted neighbor-joining (UNJ) method represents the clustering pattern of 63 genotypes in response to 17 simple sequence repeats. Genotypes are grouped into three major clusters. Cluster 1 grouped 18 accessions (blue), cluster 2 grouped 40 genotypes (red), and cluster 3 grouped 5 genotypes (green).

The population structure of the genotypes was assessed using the model-based approach program Structure 2.3.4. The peak plateau of ad hoc measure ΔK was found to be K = 2 with ΔK value of 388.18 (Fig. 3a). The model-based approach classified the genotypes into two sub-populations (SP1 and SP2) and the ancestry threshold of >65% was considered as pure, while <65% was considered as admixtures (Fig. 3b). Out of 63 genotypes studied, 27 were grouped in SP1, 22 were classified in SP2, and 14 were placed in the admixture population (Table S5). Thus, SP1 covered three moderately resistant accessions, 20 moderately susceptible accessions, one highly susceptible accession, and three susceptible accessions, while SP2 contained 20 MS accessions and two susceptible accessions. Conversely, admixtures comprised six MR and eight MS accessions. The two populations' fixation index (Fst) values were 0.3262 and 0.5525 for SP1 and SP2, respectively. Maximum allele frequency divergence between the populations was 0.2015 based on net nucleotide distance computed using point estimates of P. The average distance (expected heterozygosity) among the individuals in the panel population was 0.2697 and 0.1806 for SP1 and SP2, respectively.

**Fig. 3:**
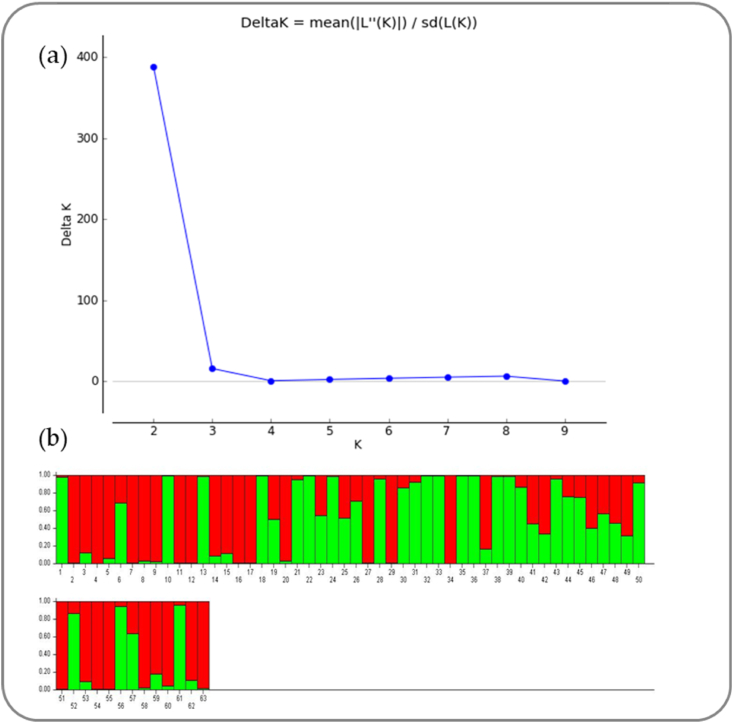
**(a)** Graph of ΔK value and ad hoc statistics related to the rate of change in the log probability of data between successive K values; **(b)** distribution pattern of 63 rice germplasm accessions based on SSRs with respective sub-populations.

#### 3.1.4 Diverse rice germplasm assignment test

The set of 63 diverse rice genotypes was grouped into two sub-populations based on phenotypic data observed from the field experiment. Twenty-three genotypes of population 1 exhibited moderate resistance against *R. solani*, while the remaining 40 genotypes exhibited moderate susceptibility and a completely susceptible reaction to sheath blight. The allelic pattern of 17 microsatellite markers distributed across the genome was used to assign the populations using GenAIEx software. Out of 23 genotypes phenotypically assigned to population 1, the population assignment test using genotypic data revealed that only 11 (47.82%) genotypes belonged to population 1; the other 12 (52.17%) were grouped into population 2. Similarly, in population 2, 21 (52.5%) out of 40 accessions were of population 2, whereas 19 (47.5%) were assigned to population 1. Thus, among the 63 accessions used in this study and compared based on phenotypic and genotypic data, 51% (32 genotypes) showed similar sub-population grouping and 49% (31) of the genotypes showed different population groupings. Hence, the selected SSRs distinguished the moderately resistant populations from the moderately susceptible and susceptible genotypes (Fig. 4) in our study.

**Fig. 4:**
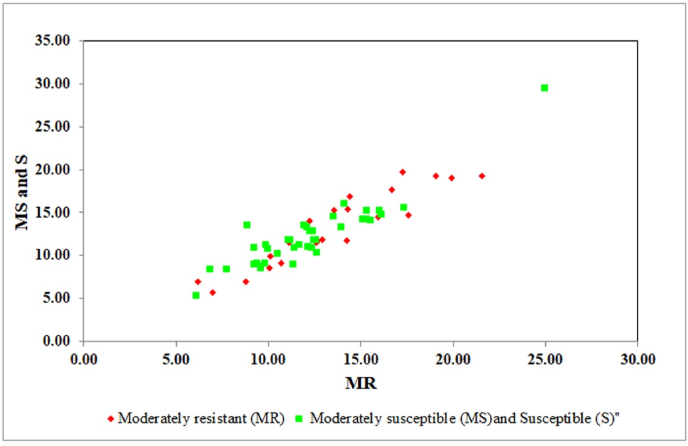
Population assignment of panel population signifying the log-likelihood assignment of each genotype using disease reaction.

#### 3.1.5 Association between morphological and sheath blight-related traits

**Fig. 5:**
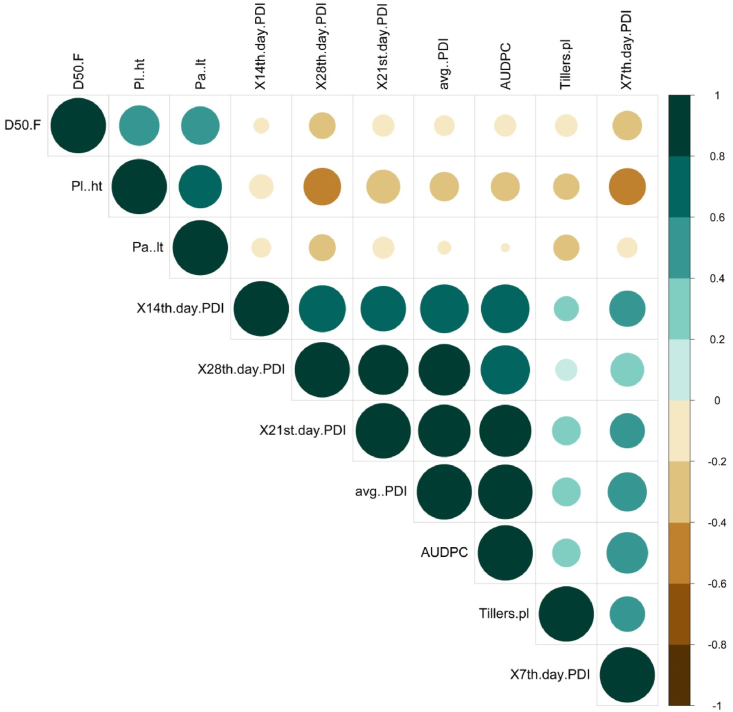
Pearson correlation among the traits associated with sheath blight disease over the season. Color (green: positive correlation; brown: negative correlation) intensity and the size of the circle are proportional to the correlation coefficient. Note: Pl. ht: plant height, D50F: days to 50% flowering, Pa. lt: panicle length, AUDPC: area under the disease progress curve, avg. PDI: average percent disease index, X7th PDI: PDI of 7th day, X14th PDI: PDI of 14th day, X21st PDI: PDI of 21st day, X28th PDI: PDI of 28th day, Tillers Pl: number of tillers per plant.

The estimated correlation coefficient among the morphological traits and traits responsible for sheath blight resistance of the wet season of 2017 is depicted in Table S3. The correlation between plant height and panicle length (0.701**, P < 0.01) and days to 50% flowering (0.477**, P < 0.01) was found to be positive. On the other hand, PDI on the 28th day (−0.661**, P < 0.01), average PDI (−0.612**, P < 0.01), 21st day PDI (−593**, P < 0.01), and AUDPC (−0.564**, P < 0.01) were observed to be negatively associated with plant height in the wet season of 2017 (Table S3). In contrast, AUDPC was strongly related to mean PDI and PDI on the 21st day, 14th day, and 28th day (0.988**, 0.946**, 0.874**, and 0.869**, P < 0.01 respectively), and mean PDI had a strong positive association with PDI of the 28th day (0.935**, P < 0.01), 21st day (0.920**, P < 0.01), and 7th day (0.835**, P < 0.01) in the wet season of 2018. In the wet season of 2018 (Table S4) and pooled over the season, AUDPC exhibited a strong positive correlation with mean PDI and PDI on the 7th day, 14th day, 21st day, and 28th day, and mean PDI was positively associated with PDI of the 28th day, 21st day, 14th day, and 7th day (Fig. 5).

### 3.2 Effect of weather parameters on sheath blight disease incidence

**Fig. 6:**
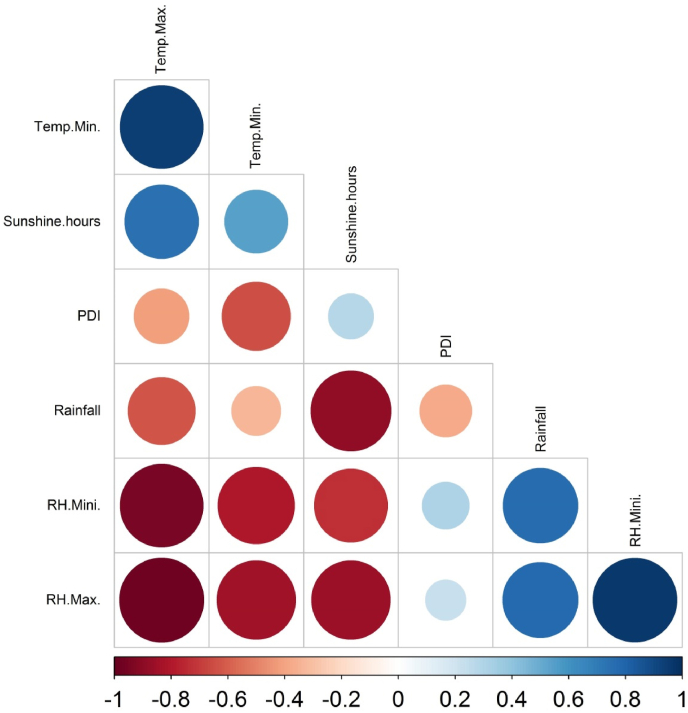
Pearson correlation among weather parameters and disease incidence during wet season 2017. Color (blue: positive correlation; red: negative correlation) intensity and the size of the circle are proportional to the correlation coefficient. Note: Temp. Max.: maximum temperature, Temp. Min.: minimum temperature, PDI: percent disease index, RH. Min.: minimum relative humidity, RH. Max.: maximum relative humidity.

**Fig. 7:**
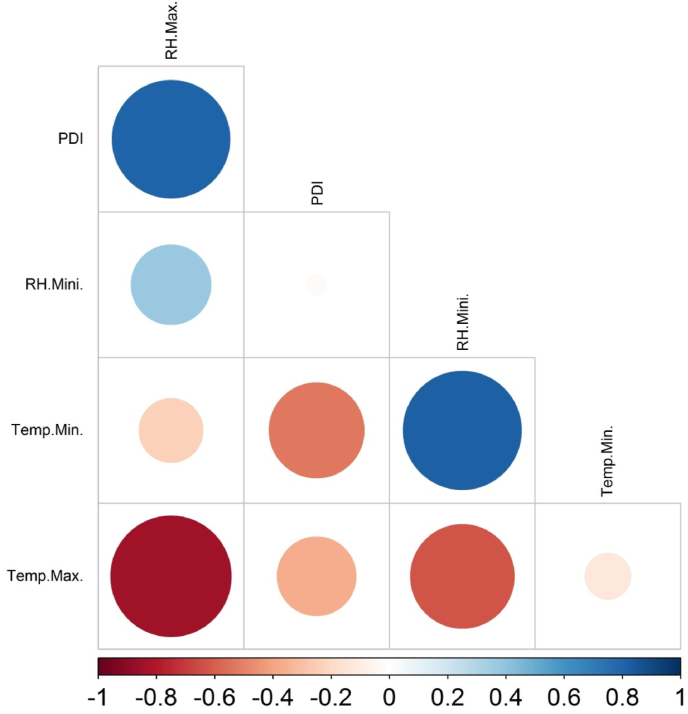
Correlation among weather parameters and disease incidence during wet season 2018. Color (blue: positive correlation; red: negative correlation) intensity and the size of the circle are proportional to the correlation coefficient. Note: Temp. Max.: maximum temperature, Temp. Min.: minimum temperature, PDI: percent disease index, RH. Min.: minimum relative humidity, RH. Max.: maximum relative humidity.

The weekly weather parameters such as temperature (maximum and minimum), rainfall, morning and evening relative humidity, and sunshine hours were correlated with weekly disease severity data of the wet season of 2017 and 2018. The PDI of both seasons had a significantly negative association with minimum (−0.643 and −0.526) and maximum temperature (−0.417 and −0.361), respectively (Fig. 6, Fig. 7). During pathogen inoculation and disease assessment, the plants received 32 mm of total rainfall in 2017 and nil in 2018. Therefore, a negative association (−0.373) was observed between PDI and rainfall in the first season (wet season 2017) of the experiment. In both seasons, PDI had a positive association with relative humidity. Notably, the maximum relative humidity (0.805) of the wet season of 2018 exhibited a very strong association with PDI.

### 3.3 Biochemical studies

In net house conditions, six defense and antioxidant-related enzymes were observed in six genotypes. ANOVA revealed significant variation across genotypes (G) and time (T) of sample collection (P > 0.01) for the defense and antioxidant enzymes studied (Table 5). Although variation was observed among the genotypes and time intervals of sample collection for change in the activity of enzymes, G × T interactions were non-significant for PPO and PAL.

**Table 5:** Analysis of variance (ANOVA) for different biochemical parameters in six rice genotypes.

Source of variation	DF	Mean sum of squares					
		PO	PPO	PAL	CAT	SOD	TP
Genotypes (G)	5	13.62**	6.75**	14.61**	12.51**	10.54**	5.76**
Time (T)	4	5.18**	2.31**	1.95**	6.06**	2.55**	2.36**
G x T	20	0.15**	0.04^ns^	0.02^ns^	0.92**	0.27**	0.12**
Error	60	0.01	0.02	0.01	0.02	0.01	0.01

** -significance value at 0.01%, ns-non-significance.

#### 3.3.1 Activity of defense-related enzymes

**Fig. 8:**
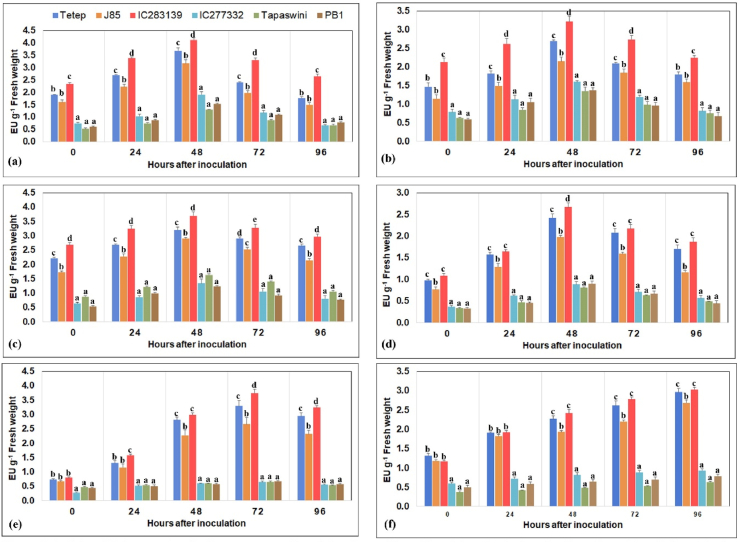
The activity of defense and antioxidant enzymes in resistant and susceptible genotypes against sheath blight disease in rice: (a) activity of peroxidase (PO), (b) activity of polyphenol oxidase (PPO), (c) activity of phenylalanine ammonia-lyase (PAL), (d) activity of total phenol, (e) activity of catalase (CAT), and (f) activity of superoxide dismutase (SOD). Bars indicate the standard error. Bars with different letters are significantly different at P < 0.05.

Results of peroxidase activity revealed that increased activity of PO was maximum at 48 h and subsequently started to decline at 72 and 96 h after pathogen inoculation. Genotype IC283139 showed maximum PO activity at 48 h, followed by Tetep and Jasmine 85. The least accumulation was found in Tapaswini and PB1 (Fig. 8a). Similarly, PPO, PAL, and total phenol activity gradually increased from 24 h after inoculation and reached a peak at 48 h, followed by a decline in activity during further time course of the experiment (Fig. 8b–d). Tolerant genotype IC283139 showed the maximum activity of PPO, PAL, and total phenol, followed by Tetep. Genotype IC283139 recorded the highest PPO activity of 3.21 EU g^−1^ FW in 48 h, followed by Tetep (2.69 EU g^−1^ FW) and Jasmine 85 (2.15 EU g^−1^ FW), and the lowest PPO activity was found in Tapaswini (1.34 EU g^−1^ FW) and PB1 (1.37 EU g^−1^ FW) (Fig. 8b). PAL activity differed from 3.69 to 1.23 μ moles of cinnamic acid min^−1^ mg^−1^ protein in the *R. solani*-inoculated genotypes used in this experiment. Genotype IC283139 recorded the maximum PAL activity (3.69 EU g^−1^ FW) at 48 h, followed by Tetep (3.20 EU g^−1^ FW) (Fig. 8c). Similarly, total phenol content was higher in resistant genotypes and lower in susceptible genotypes (Fig. 8d).

#### 3.3.2 Antioxidative enzyme activity in selected genotypes

*Rhizoctonia solani* infection substantially elevated the activity of catalase (CAT). In general, CAT activity was found to increase from 24 h after inoculation of the pathogen, attaining its peak at 72 h after inoculation; after that, it declined (Fig. 8e). The maximum CAT activity was noticed in IC283139 (3.23 EU g^−1^ FW) and Tetep (2.94 EU g^−1^ FW), followed by Jasmine 85 (2.31 EU g^−1^ FW), and the lowest CAT activity was recorded in Tapaswini (0.53 EU g^−1^ FW), PB1 (0.57 EU g^−1^ FW), and IC277332 (0.56 EU g^−1^ FW). The response of superoxide dismutase activity in rice genotypes after *R. solani* infection was notably higher in resistant cultivars than in susceptible cultivars (Fig. 8f). SOD activity was markedly elevated at 24 h after pathogen inoculation and increased continuously up to 96 h. The maximum SOD activity was observed in IC283139 (3.09 EU g^−1^ FW), followed by Tetep (2.96 EU g^−1^ FW), at 96 h after inoculation of *R. solani*, and the least was observed in Tapaswini (0.63 EU g^−1^ FW), followed by PB1 (0.78 EU g^−1^ FW).

#### 3.3.3 Genotype by enzyme biplot and association between the enzyme and sheath blight resistance in rice

**Fig. 9:**
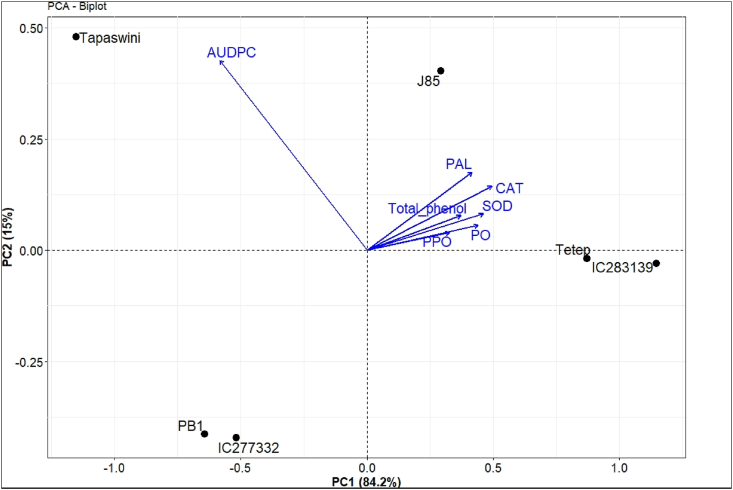
Biplot graph depicting selected genotypes in two main principal components for enzymes and disease incidence of *R. solani*. The two PC axes together explained 99.2% of the total variation. The biplot separated genotypes into two groups based on the level of tolerance. Note: AUDPC: area under the disease progress curve, PAL: phenylalanine ammonia-lyase, CAT: catalase, SOD: superoxide dismutase, PO: peroxidase, PPO: polyphenol oxidase.

The genotype by defense/antioxidant enzyme biplot generated between six genotypes and enzyme activity is presented with two principal components in Fig. 9. PC1 showed 84.2% variation, while PC2 explained 15% of the variation toward total variability. Among the different variables (enzymes and AUDPC) used to understand the role of defense enzymes in the tolerant genotypes against sheath blight, all the enzyme vectors were plotted together while the vector of AUDPC was found to be segregated away. The trait AUDPC, which was plotted in the neighboring quadrant, exhibited negative correlation with the enzymes studied, such as PO (r = −0.622), PPO (r = −0.630), PAL (r = −0.454), TP (r = −0.583), SOD (r = −0.608), and CAT (r = −0.542). Among the vectors of all the traits, the vector length of AUDPC was observed to be highly variable in our study.

Further, tolerant lines exhibited minimum AUDPC (467.96), while susceptible genotypes had maximum value (1490.74). The genotype by enzyme biplot graph (Fig. 9) clearly segregated the six genotypes into two groups: tolerant and susceptible. The two known tolerant genotypes (Tetep and Jasmine 85) and the novel tolerant genotype (IC283139) were plotted on the right side of the plot with vectors of the enzyme studied that increased the activity of defense and antioxidant enzymes. In contrast, the genotypes with a decrease in these defense and antioxidant enzymes with higher AUDPC values were plotted on the opposite quadrant of the tolerant genotypes.

#### 3.3.4 Effect of weather parameters on defense and antioxidant enzymes

**Fig. 10:**
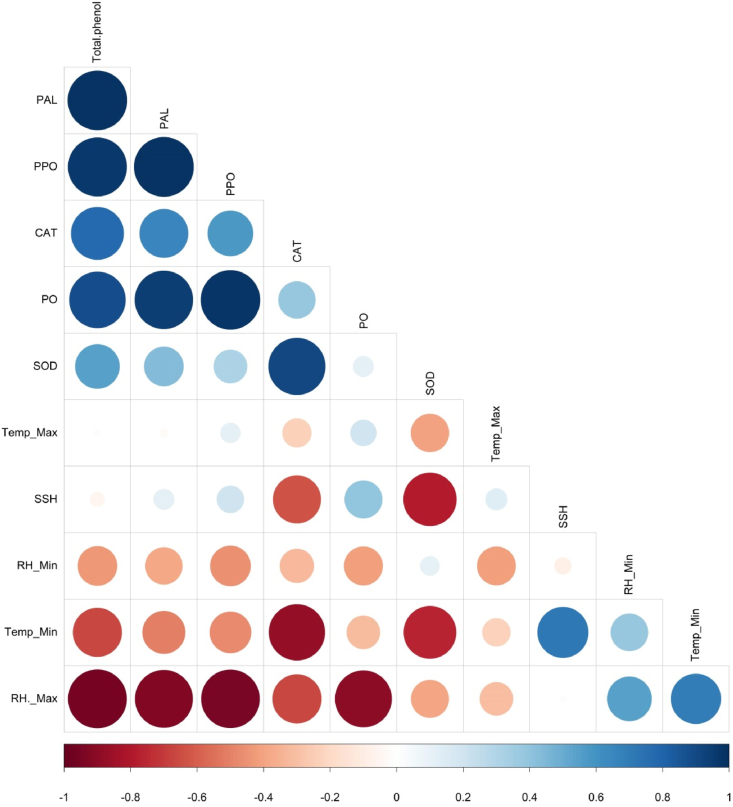
Pearson correlation among defense and antioxidant enzymes with weather parameters. Color (blue: positive correlation; red: negative correlation) intensity and the size of the circle are proportional to the correlation coefficient. Note: PAL: phenylalanine ammonia-lyase, CAT: catalase, SOD: superoxide dismutase, PO: peroxidase, PPO: polyphenol oxidase, Temp. Max.: maximum temperature, Temp. Min.: minimum temperature SSH: sunshine hours, RH. Min.: minimum relative humidity, RH. Max.: maximum relative humidity.

The estimated Pearson correlation coefficient among the enzymes and weather parameters against *R. solani* is depicted in Fig. 10. A highly positive correlation was found between peroxidase and polyphenol oxidase (r = 0.976), phenylalanine ammonia-lyase (r = 0.941), and total phenol (r = 0.882). Similarly, PPO exhibited a strong association with PAL (r = 0.988) and total phenol (r = 963). Among the antioxidant enzymes, superoxide dismutase had a high positive correlation with catalase (r = 0.901). On the other hand, a positive association was also observed between CAT and total phenol (r = 0.772). With regards to weather parameters, a strong negative association was observed between minimum temperature and expression of CAT (r = −0.875). Additionally, maximum relative humidity had also registered a strong negative association with total phenol (r = −0.957), PPO (r = −0.949), PAL (r = −0.926), and PO (r = −0.894) activities (Fig. 10).

## 4 Discussion

Quantitative resistance has been associated with long-term and broad-spectrum resistance, making this a valuable resource for rice breeding. Sheath blight resistance in rice conferring quantitative resistance is controlled by multiple genes [4], with each gene having a small effect on the trait. So far, a sizable number of rice genotypes were found to be moderately resistant to sheath blight: Tetep, Jasmine 85, Teqing, Minghui, ZYQ8, WSS2, Tadukan, LSBR-5, LSBR-33, ARC10531, SM801, Ngnololasha, Wazuhophek, Gumdhan, and Phougak [5]. Among them, Tetep [5], Jasmine 85 [7], and Teqing [5] were widely used as checks for sheath blight disease evaluation studies in rice and also for the identification of QTLs for sheath blight disease resistance. In contrast, rice cultivars such as Lemont, HP2216, Pusa Basmati-1, Tapaswini, Zhenshan97B, Hinohikari, Rosement, BPT 5204, and Koshikari were reported as highly susceptible to sheath blight of rice [6,14] and extensively used as susceptible checks. In our study, 63 genotypes, including landraces and improved lines of *indica*, were screened for sheath blight resistance. Among them, 23 genotypes were moderately resistant, 38 were moderately susceptible, and 2 exhibited a susceptible reaction to sheath blight disease. The population structure of this germplasm was differentiated into two sub-populations (49 accessions were pure and 14 were admixture). The admixture comprised six moderately resistant genotypes and eight moderately susceptible ones (Table S5).

Interestingly, our two years of rigorous screening revealed that the positive checks such as Jasmine 85 and Teqing exhibited moderately susceptible reactions. In contrast, earlier researchers reported Teqing [5] and Jasmine 85 [7] as moderately resistant to sheath blight disease in rice. Our results agree with the findings of Dey et al. (2016), who had noticed Jasmine 85 as susceptible to *R. solani* in their three years of consecutive screening. In addition, Tetep was found to be moderately resistant against sheath blight, and the same was reported by Dey et al. [5] and Goswami et al. [6]. The two years of screening yielded better results in identifying moderately resistant genotypes such as IC283139, IC283041, IC283038, and IC283023, which exhibited less disease reaction than positive check Tetep. These findings encouraged us to further investigate the nature of resistance before using these genotypes in the breeding program. Earlier reports showed that agro-morphological traits such as plant height, lower number of tillers, and late flowering were positively correlated with a delay in disease development [5]. Similarly, our results showed that genotypes with a lower tiller number, late flowering, and increased plant height were moderately resistant to sheath blight.

Further, to understand the relatedness between genotypes at the genotypic level and the ability of primers that distinguish genotypes based on the level of tolerance, we used 17 microsatellite markers and they generated 54 alleles with a mean value of 3.176 alleles per marker and a mean PIC value of 0.427. Our results were in accordance with a previous report of Pradhan et al. [34], who also observed an average of alleles per locus of 2.44, with 1–6 alleles per locus, and PIC values ranging from 0.000 to 0.671, with a mean of 0.355, in the collection of rice accessions including landraces and improved varieties. The mean value of the major allele frequency was observed to be 0.612 per marker, while gene diversity was 0.491 per locus. These results were in agreement with the earlier reports of Anandan et al. [35] and Yadav et al. [36]. Further, the distance-based neighbor-joining tree generated out of genotypic data grouped the panel population into three major clusters. Cluster 1 comprises one each of moderately resistant and susceptible genotypes and 16 moderately susceptible genotypes, with an average PDI of 24.04, and cluster 2 grouped 6, 29, 4, and 1 genotype(s) of MR, MS, S, and HS, respectively, with a mean PDI of 24.55. Further, cluster 3 comprised two MR and three MS genotypes, with an average PDI of 19.74. Among the three clusters, clusters 1 and 2 had an average PDI of about 24, whereas cluster 3 had an average PDI of 19.74. In addition, model-based population structure analysis confirms the results of distance-based cluster analysis. Among the three sub-populations (SP1, SP2, and AD), SP1 and SP2 had an average PDI of 25.0 and population AD had an average PDI of 20.25. In addition, the population assignment test grouped the 63 accessions into two proportions in a similar manner. Therefore, the SSRs used in this study have distinguished resistant populations from susceptible populations.

Plants synthesize several aromatic compounds consisting largely of phenols or their derivatives [37] against biotic and abiotic stress factors to protect themselves. These compounds play an important role in activating the plant defense and antioxidant mechanism against infections by various pathogens. Thus, understanding the defense and antioxidant activities conferring resistance against *R. solani* could offer valuable information on host-pathogen interaction in rice. The findings of our study demonstrated that the activities of PO and PPO significantly increased throughout the study period (96 h) in resistant rice genotypes (IC283139 and Tetep) more than in susceptible genotypes (Tapaswini and PB-1) (Fig. 8a and b). Peroxidase and PPO help the plants catalyze lignin formation and play a vital role in resistance to pathogens. Similarly, Mondal et al. [9] and Pavani et al. [38] also reported the activity of defense enzymes against sheath blight in rice. However, Mondal et al. [5,9] reported the activity of defense and antioxidant enzymes in a single rice genotype (MTU-7029), while the level of enzyme activity was reported by Pavani et al. [38] was found to be less than in our study. Moreover, PO is involved in the reduction of ROS and catalyzes the oxido-reduction of different substrates using H_2_O_2_ [39]. In higher plants, the first step of PAL catalyzes the biosynthesis of the phenylpropanoid pathway, which is involved in offering resistance to plants against fungal plant pathogens [40]. In our study, the activity of PAL recorded a maximum in resistant cultivars and a comparative minimum in susceptible cultivars (Fig. 8c). In addition to PO, PPO, and PAL, the accumulation of phenol and its derivatives in plants or their synthesis in response to the invasion of pathogens is associated with resistance, and an increase in phenolic content in the plant system has often been correlated with increased resistance to biotic stress [41]. It is well known that resistant plants more promptly obtain a high level of phenols or polyphenols than susceptible plants. In our study, resistant genotypes induced a higher accumulation of phenolic compounds than susceptible genotypes, and this increased concentration might augment the resistance to sheath blight disease in rice. Our results are in accordance with those of earlier reports by Gupta et al. [42].

Reactive oxygen species (ROS) are the second most important messenger molecule in plants that enables defense-related genes and affects other major signaling molecules [43]. The unrestrained accumulation of ROS resulted in increased cell death, which enhances plant susceptibility [44]. Antioxidant enzymes such as CAT and SOD were involved in scavenging different types of ROS by catalyzing the dismutation of O^2^ to O_2_ and H_2_O_2_ [39,43]. In our study, both CAT and SOD accumulation were significantly higher in infected sheaths of resistant cultivars and lower in susceptible cultivars (Fig. 8e and f). A similar result was previously reported by Mohapatra et al. [42].

**Fig. 11:**
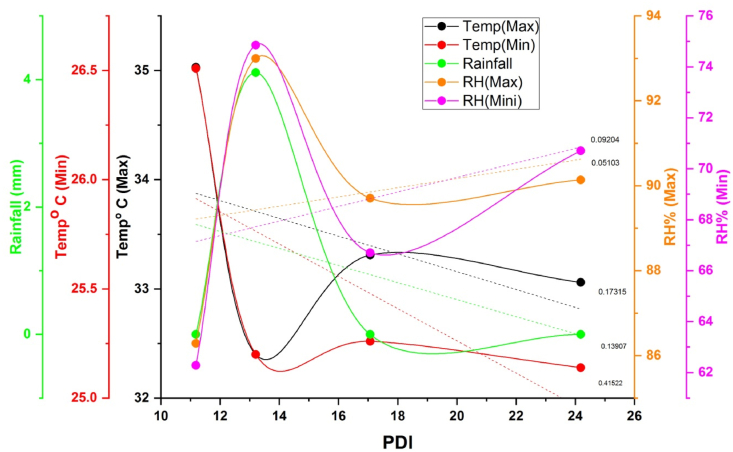
Effect of weather parameters on sheath blight disease development during wet season 2017. Note: Temp (Max): maximum temperature, Temp (Min): minimum temperature, RH (Max): maximum relative humidity, RH (Min): minimum relative humidity. PDI: percent disease index.

**Fig. 12:**
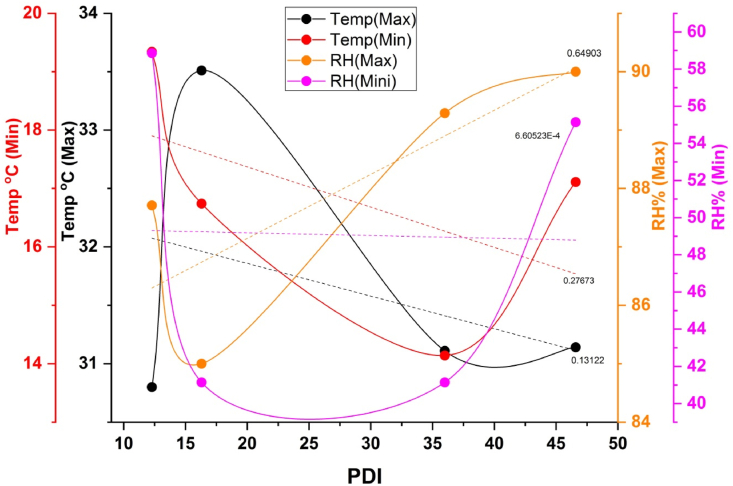
Effect of weather parameters on sheath blight disease development during wet season 2018. Precipitation was not observed during the period of pathogen inoculation and disease assessment in wet season 2018. Note: Temp (Max): maximum temperature, Temp (Min): minimum temperature, RH (Max): maximum relative humidity, RH (Min): minimum relative humidity. PDI: percent disease index.

**Fig. 13:**
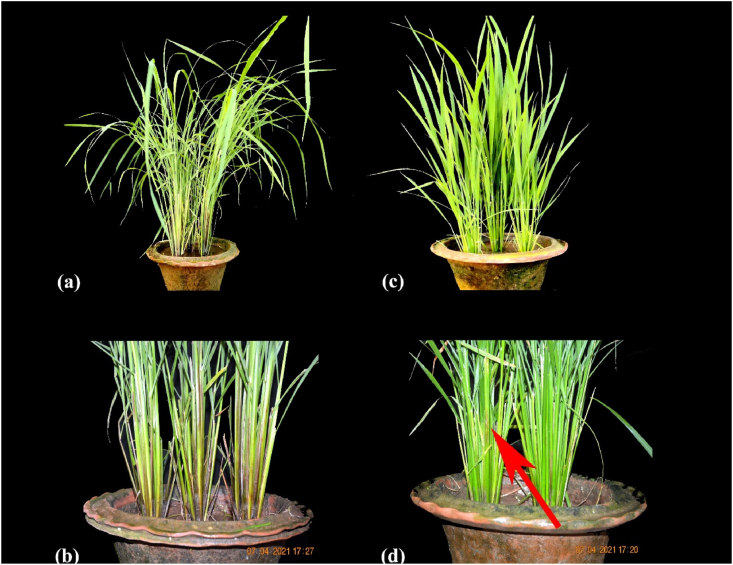
Disease reaction of susceptible and tolerant genotypes against sheath blight disease 24 h after inoculation: (a) tolerant genotype IC283139 at the vegetative stage, (b) tolerant genotype IC283139 with purple base without any symptoms, (c) susceptible genotype Tapaswini at the vegetative stage, and (d) susceptible genotype Tapaswini. *R. solani* infects rice leaf sheaths at the base of the culm.

Weather parameters play a vital role in the development and progression of sheath blight disease in rice. In recent days, more emphasis has been given to weather-based forecasting models for the prediction of disease outbreaks. Earlier findings indicated that maximum relative humidity and minimum temperature played a vital role in disease initiation [45]. Similarly, our results also suggested a similar role of weather parameters in disease progression. The negative association between PDI and maximum temperature (R^2^ of the wet season of 2017: 17%; wet season of 2018: 13%), minimum temperature (R^2^ of the wet season of 2017: 41%; wet season of 2018: 27%), or rainfall (R^2^ of the wet season of 2017: 13%) and the positive association with maximum relative humidity (Fig. 11, Fig. 12) suggest that very low temperature or high precipitation might have a negative effect on pathogen establishment. Our results agreed with Pal et al. [45], who reported maximum temperature from 31 °C to 34 °C and minimum temperature from 17 °C to 23 °C, coupled with 70%–80% relative humidity, which was a conducive environment for the development and spread of sheath blight disease. However, the report of Pal et al. [44] was based on a single genotype over two seasons. Our study's findings seem to be the first report on the effect of weather parameters on the incidence of sheath blight disease using 63 rice germplasm accessions under artificial inoculation in open field conditions. On the other hand, the strong positive association with maximum relative humidity (R^2^ of the wet season of 2017: 5%; wet season of 2018: 64%) and the absence of rain during the 28 days of the disease observance period in the wet season of 2018 might assist the pathogen in the rapid establishment. Similarly, Hashiba et al. [46] observed that initiation of sheath blight disease was most prevalent at a temperature of 28 °C and relative humidity of 100% along with continuous low precipitation during the disease development period. These findings were established based on the average AUDPC data from the wet season of 2017 (480.76) and 2018 (798.13) (Table 2, Table 3) and the classification of genotypes based on disease reaction (Figs. S1 and S2). Therefore, the wet season of 2018 average maximum relative humidity of 88%, the maximum temperature of 31.6 °C, and the minimum temperature of 16.8 °C might favor the establishment of a pathogen faster. The non-significant AUDPC at G x S and 7th day PDI at both the genotypic level and G x S showed that innate immunity plays a significant role in sheath blight resistance. The non-significant PDI on the 7th day at the genotypic level suggests that initiation of diseases is based on prevailing conducive environmental conditions for the pathogen and the non-significant AUDPC at G x S suggests that innate immunity (defense and antioxidant enzymes) of the genotype acts against the pathogen after infection and stops further establishment of the pathogen in tolerant genotypes. Therefore, understanding the role of environmental factors with defense-related enzymes is vital for controlling sheath blight disease. Further, the correlation between the enzymes and disease severity or AUDPC confirmed the significance of enzymes involved in a resistant reaction against sheath blight. Interestingly, all the enzymes studied exhibited a strong association between them and a negative association with AUDPC. This clearly demonstrated the role and accumulation of defense and antioxidant enzymes in disease reaction according to their level of resistance or susceptibility to a pathogen. Our findings were closely associated with those of Mohapatra et al. [46], wherein they reported that AUDPC had a high negative association with enzymes. However, the negative association between enzymes and maximum relative humidity suggests that low temperature affects the synthesis of sufficient enzymes to have tolerance against sheath blight. To support this hypothesis, the tolerant genotype IC283139 has a purple base (Fig. 13a&b) and is found to be better than positive check Tetep with a higher level of enzymes. Finding the association between weather parameters and the enzyme activity of defense and antioxidant enzymes with challenge inoculation of *R. solani* at different time intervals in our work has not been reported so far to our knowledge. The accumulation of anthocyanin was reported to have been associated with resistance to low-temperature stress [47,48]. In addition, Zhang et al. [49] observed that the Chl fluorescence value of Fv/Fm was not significantly decreased in plants with high anthocyanin content, and the accumulation of H_2_O_2_ and superoxide anions was relatively low in purple-pigmented plants treated at 4 °C. Therefore, the anthocyanin in the stem base of the tolerant genotype IC283139 might have offered a protective role in synthesizing a higher amount of enzymes under minimum temperature.

## 5 Conclusions

In our investigation, novel and alternate sources for sheath blight resistance have been identified and this helped elucidate the role of maximum relative humidity in its negative effect on defense enzyme production. Further, the mechanism of host-pathogen interaction showed genotypes with higher variation for antioxidant defense responses between resistant and susceptible genotypes. The moderately resistant genotypes such as IC283139 and Tetep exhibited a higher level of defense and antioxidant enzymes. The reverse trend was observed in the case of susceptible genotypes at different times of pathogen inoculation. These observations suggest that activation of induced systemic resistance and antioxidant defense response makes resistant genotypes less predisposed to oxidative damage caused by *R. solani* inciting sheath blight disease. The identified novel genotype (IC283139) could serve as an alternate source of resistance and should be taken forward to identify the QTLs and genes involved in resistance to develop rice varieties with sheath blight resistance. In addition, studies on functional genomics and transcriptomics could offer more insight into the mechanism of the resistance against sheath blight involved in this novel tolerant genotype.

## Author statement

**Conceptualization:** A.A., N.R., V.S., and B.C.P. **Conducting of the experiment and the methodology:** N.R., S.R.P., G.S., U.K., P.K.S., and A.A. **Data curation, phenotypic trait characterization, data analysis, and validation:** N.R., S.R.P., G.S., U.K., P.K.S., P.C., and A.A. **Writing (original draft preparation):** N.R., S.R.P., G.S., U.K., P.K.S., A.M., P.C., and A.A. **Writing (review and editing):** N.R., S.R.P., V.S., G.S., U.K., P.K.S., A.M., J.A., and A.A. **All authors read and approved the final manuscript.**

## Funding

This research was funded by the University Grants Commission, New Delhi, India, that provided a research fellowship to N.R. for pursuing his Ph.D program; the 10.13039/100000865Bill & Melinda Gates Foundation (BMGF) for providing a research grant to the Green Super Rice Project under ID OPP1130530.

## Institutional review board statement

Not applicable.

## Informed consent statement

Not applicable.

## Declaration of competing interest

The authors declare that they have no known competing financial interests or personal relationships that could have appeared to influence the work reported in this paper.

## Figures and Tables

**figs1:**
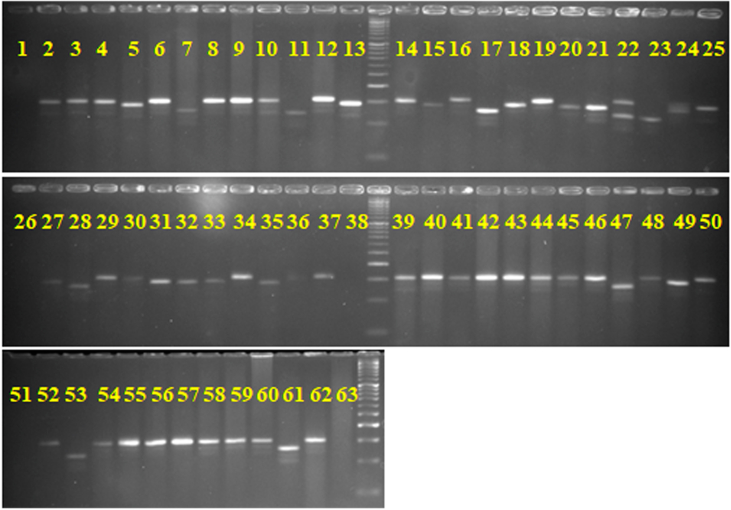


**figs2:**
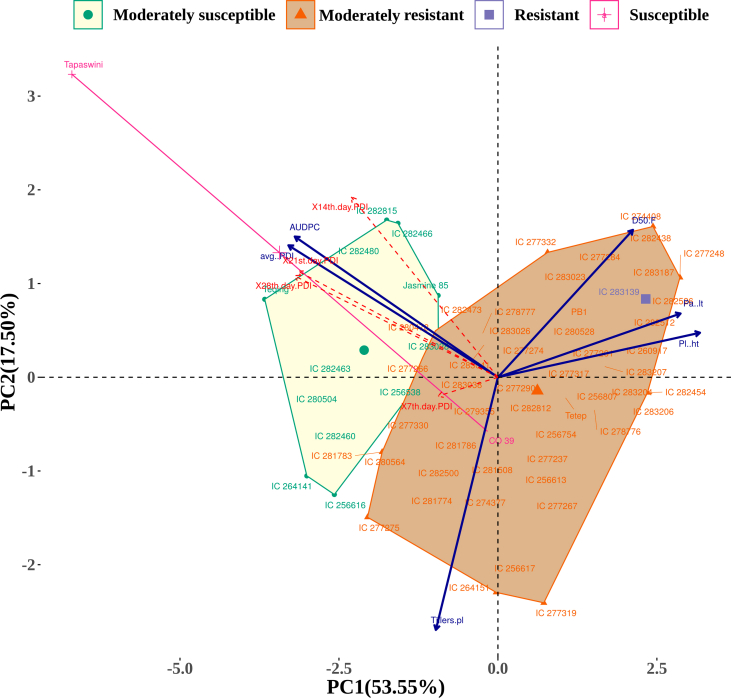


**figs3:**
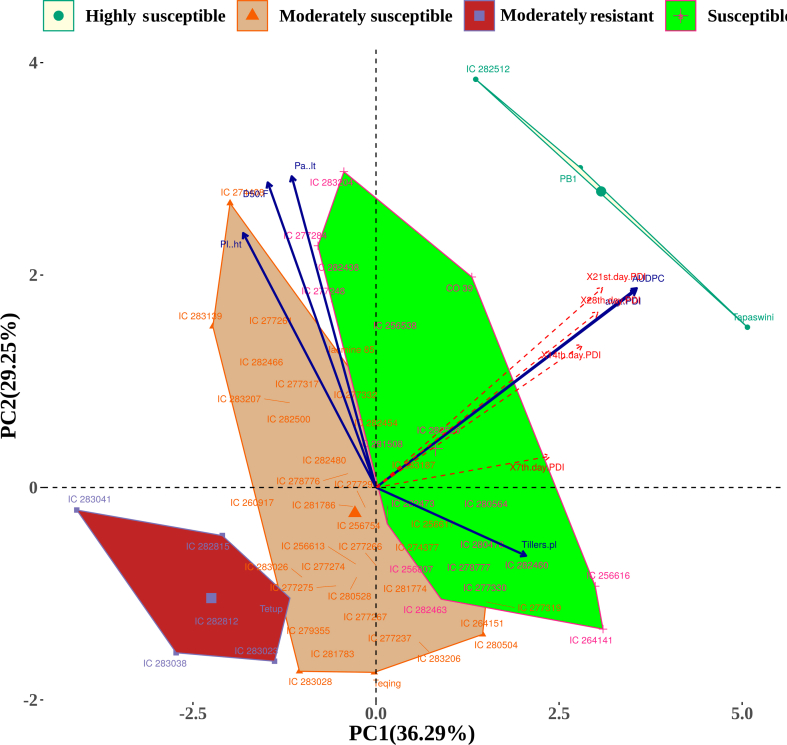


**figs4:**
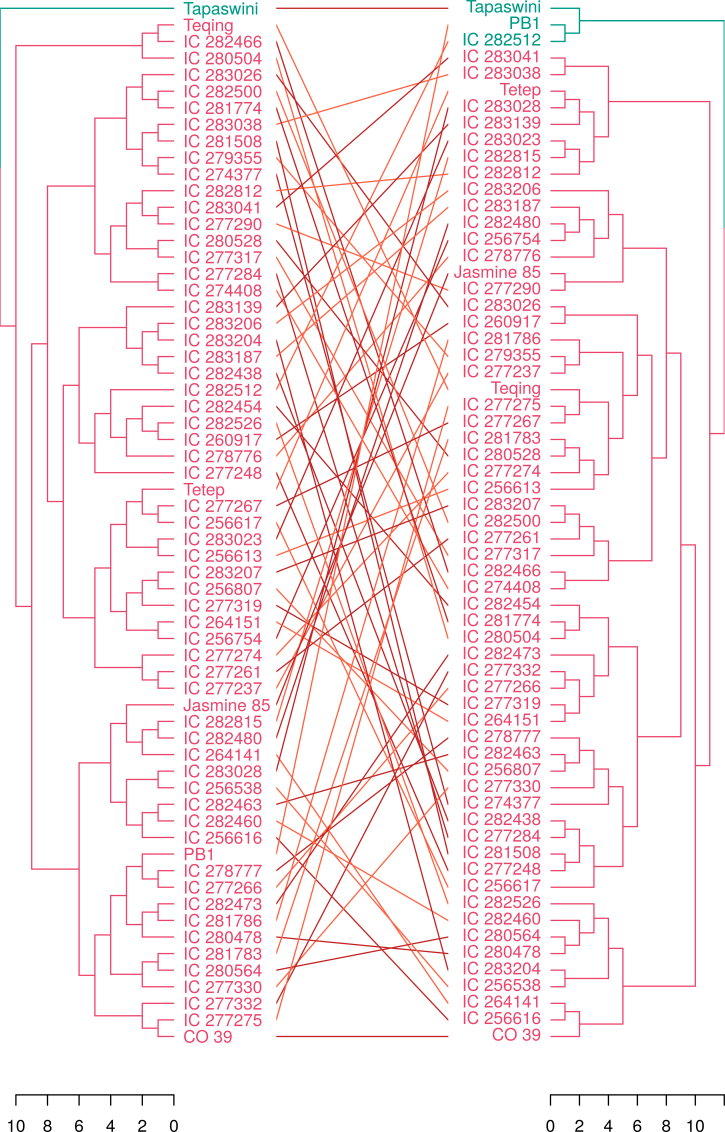


## Data Availability

Data will be made available on request.
