# Supplementary material for: Deciphering environmental factors and defense response of rice genotypes against sheath blight disease
Source: Physiol Mol Plant Pathol. 2022 Nov;122:101916. doi: 10.1016/j.pmpp.2022.101916 (PMC9669783; doi:10.1016/j.pmpp.2022.101916)
Supplement: Multimedia component 1 [file mmc1.docx]

**Table S1 Details of rice germplasm used for this study.**

| **Sl. No** | **Genotypes** | **Collection** | **Plant Height** | **Panicle Length** | **No of tillers/Plant** | **Days to Heading** |
| --- | --- | --- | --- | --- | --- | --- |
| 1 | IC 277237 | IAS, BHU | 89.85±10.54 | 19.64±1.04 | 11.30±3.11 | 106.00±8.49 |
| 2 | IC 277317 | IAS, BHU | 119.07±8.39 | 26.17±0.45 | 11.30±0.85 | 103.50±2.12 |
| 3 | IC 277319 | IAS, BHU | 113.24±26.25 | 21.12±3.16 | 19.70±1.56 | 99.00±2.83 |
| 4 | IC 278776 | IAS, BHU | 128.67±34.69 | 23.03±1.53 | 8.95±1.06 | 98.50±4.95 |
| 5 | IC 282454 | IAS, BHU | 120.32±56.26 | 27.97±1.30 | 9.60±2.26 | 102.00±2.83 |
| 6 | IC 256613 | IAS, BHU | 110.39±16.00 | 21.49±2.08 | 12.20±2.12 | 100.00±1.41 |
| 7 | IC 256616 | IAS, BHU | 71.41±0.55 | 19.21±2.50 | 15.05±1.34 | 89.50±12.02 |
| 8 | IC 256617 | IAS, BHU | 106.08±16.43 | 21.28±1.93 | 17.00±2.69 | 102.50±0.71 |
| 9 | IC 256754 | IAS, BHU | 111.29±7.38 | 21.79±2.66 | 9.50±0.85 | 94.50±3.54 |
| 10 | IC 256807 | IAS, BHU | 100.10±39.18 | 21.98±2.26 | 8.15±0.64 | 100.00±2.83 |
| 11 | IC 260917 | IAS, BHU | 128.55±39.25 | 24.65±1.89 | 7.15±0.92 | 102.50±0.71 |
| 12 | IC 264141 | IAS, BHU | 62.12±7.89 | 18.47±2.02 | 13.15±2.62 | 87.50±12.02 |
| 13 | IC 264151 | IAS, BHU | 103.30±7.92 | 18.48±2.12 | 17.60±1.70 | 100.00±4.24 |
| 14 | IC 274377 | IAS, BHU | 81.15±7.85 | 21.62±1.52 | 11.95±1.06 | 99.00±7.07 |
| 15 | IC 274408 | IAS, BHU | 119.43±13.68 | 27.61±0.46 | 9.15±1.20 | 132.00±8.49 |
| 16 | IC 277248 | IAS, BHU | 131.94±44.63 | 27.74±2.18 | 5.65±0.78 | 108.50±0.71 |
| 17 | IC 277261 | IAS, BHU | 107.45±19.86 | 25.79±0.21 | 7.30±0.57 | 107.00±2.83 |
| 18 | IC 277266 | IAS, BHU | 91.47±11.69 | 17.24±2.15 | 6.05±1.34 | 98.50±4.95 |
| 19 | IC 277274 | IAS, BHU | 99.88±11.91 | 19.57±0.98 | 8.75±1.20 | 111.50±12.02 |
| 20 | IC 277284 | IAS, BHU | 121.54±38.41 | 27.35±1.94 | 4.90±0.71 | 111.00±1.41 |
| 21 | IC 277290 | IAS, BHU | 102.66±8.12 | 22.14±2.27 | 9.15±0.21 | 99.00±7.07 |
| 22 | IC 277330 | IAS, BHU | 68.20±13.16 | 21.33±2.38 | 10.20±3.11 | 96.00±8.49 |
| 23 | IC 277332 | IAS, BHU | 114.01±21.91 | 26.31±2.49 | 5.15±0.78 | 99.50±4.95 |
| 24 | IC 278777 | IAS, BHU | 77.97±7.97 | 22.05±2.29 | 8.80±0.42 | 103.00±2.83 |
| 25 | IC 279355 | IAS, BHU | 84.50±17.68 | 20.15±0.54 | 5.65±1.91 | 95.50±6.36 |
| 26 | IC 280478 | IAS, BHU | 90.51±1.70 | 17.66±0.52 | 4.80±0.99 | 95.00±4.24 |
| 27 | IC 280504 | IAS, BHU | 66.45±5.59 | 20.87±1.27 | 14.40±0.71 | 97.00±4.24 |
| 28 | IC 280528 | IAS, BHU | 103.33±15.94 | 23.92±2.45 | 9.25±0.21 | 100.50±6.36 |
| 29 | IC 280564 | IAS, BHU | 72.33±6.41 | 22.10±1.53 | 11.20±2.40 | 95.50±0.71 |
| 30 | IC 281508 | IAS, BHU | 103.34±3.90 | 23.56±3.23 | 9.40±4.81 | 93.00±11.31 |
| 31 | IC 281783 | IAS, BHU | 87.71±9.62 | 17.25±1.26 | 8.25±2.19 | 88.50±10.61 |
| 32 | IC 281786 | IAS, BHU | 81.93±12.40 | 25.75±0.26 | 10.30±1.41 | 92.50±13.44 |
| 33 | IC 282438 | IAS, BHU | 113.73±21.74 | 25.80±1.39 | 6.40±0.71 | 123.50±6.36 |
| 34 | IC 282460 | IAS, BHU | 78.98±13.69 | 20.76±0.21 | 12.90±2.12 | 90.00±2.83 |
| 35 | IC 282463 | IAS, BHU | 72.11±6.09 | 18.79±0.82 | 7.55±1.06 | 91.50±9.19 |
| 36 | IC 282466 | IAS, BHU | 97.82±4.07 | 24.97±1.50 | 8.05±0.49 | 106.50±17.68 |
| 37 | IC 282473 | IAS, BHU | 98.49±0.58 | 19.21±3.97 | 5.85±0.21 | 100.00±7.07 |
| 38 | IC 282480 | IAS, BHU | 89.82±17.71 | 21.52±0.47 | 6.55±0.64 | 99.50±6.36 |
| 39 | IC 282500 | IAS, BHU | 94.22±20.81 | 23.30±0.96 | 11.30±3.39 | 97.50±3.54 |
| 40 | IC 282512 | IAS, BHU | 129.98±21.52 | 28.81±0.55 | 6.50±1.41 | 103.00±4.24 |
| 41 | IC 282526 | IAS, BHU | 132.51±36.61 | 23.87±4.89 | 6.70±1.70 | 100.50±0.71 |
| 42 | IC 282812 | IAS, BHU | 113.14±18.47 | 23.97±0.23 | 9.85±3.61 | 94.50±3.54 |
| 43 | IC 282815 | IAS, BHU | 89.18±11.84 | 20.91±2.79 | 5.85±0.07 | 107.00±4.24 |
| 44 | IC 283023 | IAS, BHU | 108.66±19.71 | 21.72±4.04 | 8.05±3.61 | 108.50±9.19 |
| 45 | IC 283026 | IAS, BHU | 83.71±7.21 | 23.04±0.85 | 7.50±2.26 | 106.50±7.78 |
| 46 | IC 283028 | IAS, BHU | 91.12±3.65 | 21.02±5.35 | 8.90±3.54 | 97.50±3.54 |
| 47 | IC 283038 | IAS, BHU | 102.74±1.18 | 19.88±0.45 | 6.65±0.35 | 97.50±4.95 |
| 48 | IC 283041 | IAS, BHU | 112.06±4.89 | 20.62±0.75 | 6.20±0.28 | 105.50±19.09 |
| 49 | IC 283139 | IAS, BHU | 129.56±2.18 | 23.75±2.45 | 7.90±0.00 | 122.00±4.24 |
| 50 | IC 283204 | IAS, BHU | 144.21±18.12 | 26.10±0.26 | 10.00±3.96 | 123.50±4.95 |
| 51 | IC 283207 | IAS, BHU | 125.07±16.17 | 25.84±0.20 | 8.50±0.28 | 106.50±4.95 |
| 52 | IC 256538 | IAS, BHU | 109.96±36.85 | 21.41±2.23 | 11.90±0.28 | 98.50±4.95 |
| 53 | IC 277267 | IAS, BHU | 100.38±30.02 | 23.24±1.25 | 14.70±0.85 | 99.50±0.71 |
| 54 | IC 277275 | IAS, BHU | 81.74±9.71 | 20.16±1.80 | 11.55±6.43 | 89.00±5.66 |
| 55 | IC 281774 | IAS, BHU | 70.70±5.94 | 21.87±2.45 | 12.15±3.89 | 99.50±6.36 |
| 56 | IC 283187 | IAS, BHU | 114.21±26.86 | 22.97±4.00 | 9.70±1.98 | 124.00±5.66 |
| 57 | IC 283206 | IAS, BHU | 104.05±44.48 | 23.07±6.57 | 11.45±4.60 | 110.00±16.97 |
| 58 | CO 39 | ICAR-NRRI | 122.32±11.82 | 23.79±0.06 | 13.88±5.06 | 106.00±2.83 |
| 59 | Teqing | ICAR-NRRI | 75.58±13.22 | 18.44±1.41 | 10.75±0.07 | 95.00±2.83 |
| 60 | Jasmine 85 | ICAR-NRRI | 111.92±9.99 | 20.76±0.83 | 13.00±0.57 | 120.00±5.66 |
| 61 | PB1 | ICAR-NRRI | 99.91±10.59 | 29.52±1.48 | 11.65±0.35 | 111.00±4.24 |
| 62 | Tapaswini | ICAR-NRRI | 79.55±9.84 | 18.21±1.68 | 13.63±1.45 | 102.00±1.41 |
| 63 | Tetep | ICAR-NRRI | 126.33±15.37 | 21.85±1.24 | 10.25±0.21 | 99.00±5.66 |

Data followed by + standard deviation
