# Supplementary material for: Deciphering environmental factors and defense response of rice genotypes against sheath blight disease
Source: Physiol Mol Plant Pathol. 2022 Nov;122:101916. doi: 10.1016/j.pmpp.2022.101916 (PMC9669783; doi:10.1016/j.pmpp.2022.101916)
Supplement: Multimedia component 2 [file mmc2.docx]

**Table S2 Details of SSR primers used for genotyping of 63 rice genotypes against sheath blight.**

| **S. no.** | **Marker** | **Chromosome** | **Forward Primer** | **Reverse Primer** | **Annealing temperature** | **Product size** |
| --- | --- | --- | --- | --- | --- | --- |
| 1 | RM306 | 1 | CAAGGTCAAGAATGCAATGG | GCCACTTTAATCATTGCATC | 52 | 155 |
| 2 | RM5529 | 2 | AGCCGAAACTACATTCGGTG | TTGTGTAGTTGGCACGCTTC | 55 | 167 |
| 3 | RM1350 | 3 | CGCCCTAGTAGATAGGTAATTG | AAATCAGCAAGAAAGCTCTG | 56 | 167 |
| 4 | RM16 | 3 | CGCTAGGGCAGCATCTAAA | AACACAGCAGGTACGCGC | 55 | 181 |
| 5 | RM3117 | 3 | GCCATCTCTCTCTCTCTCTCTC | CCTTAGCTCATCAAGCGAGG | 55 | 111 |
| 6 | RM570 | 3 | GTTCTTCAACTCCCAGTGCG | TGACGATGTGGAAGAGCAAG | 55 | 208 |
| 7 | RM81 | 3 | GAGTGCTTGTGCAAGATCCA | CTTCTTCACTCATGCAGTTC | 58 | 110 |
| 8 | RM335 | 4 | GTACACACCCACATCGAGAAG | GCTCTATGCGAGTATCCATGG | 55 | 104 |
| 9 | RM518 | 4 | CTCTTCACTCACTCACCATGG | ATCCATCTGGAGCAAGCAAC | 55 | 171 |
| 10 | RM13 | 5 | TCCAACATGGCAAGAGAGAG | GGTGGCATTCGATTCCAG | 58 | 141 |
| 11 | RM178 | 5 | TCGCGTGAAAGATAAGCGGCGC | GATCACCGTTCCCTCCGCCTGC | 55 | 117 |
| 12 | RM334 | 5 | GTTCAGTGTTCAGTGCCACC | GACTTTGATCTTTGGTGGACG | 55 | 182 |
| 13 | RM5428 | 8 | ATGCAATACAGCACACTCGC | CTTATGCTCTCATGGCTCCC | 55 | 222 |
| 14 | RM3452 | 8 | GGCAGCCCATCAACTAGATC | TTGCAAACCCTAGTCCAAGC | 55 | 190 |
| 15 | RM257 | 9 | CAGTTCCGAGCAAGAGTACTC | GGATCGGACGTGGCATATG | 55 | 147 |
| 16 | RM3823 | 9 | CTCCTTCAGTCGGTCGTC | AAGGAGTCTGTCGCTTTACC | 55 | 192 |
| 17 | RM101 | 12 | AAGGAGTCTGTCGCTTTACC | ACACAACATGTTCCCTCCCATGC | 55 | 324 |
