# Supplementary material for: Deciphering environmental factors and defense response of rice genotypes against sheath blight disease
Source: Physiol Mol Plant Pathol. 2022 Nov;122:101916. doi: 10.1016/j.pmpp.2022.101916 (PMC9669783; doi:10.1016/j.pmpp.2022.101916)
Supplement: Multimedia component 3 [file mmc3.docx]

**Table S3 Correlation among traits associated with sheath blight disease during wet season 2017.**

| **Characters** | **Plant height** | **Panicle length** | **No. of tillers/plant** | **Days to 50% flowering** | **7th day PDI** | **14th day PDI** | **21st day PDI** | **28th day PDI** | **Average PDI** | **AUDPC** |
| --- | --- | --- | --- | --- | --- | --- | --- | --- | --- | --- |
| **Plant height** | 1.000 |  |  |  |  |  |  |  |  |  |
| **Panicle length** | 0.701** | 1.000 |  |  |  |  |  |  |  |  |
| **No. of tillers/plant** | -0.187 | -0.176 | 1.000 |  |  |  |  |  |  |  |
| **Days to 50% flowering** | 0.477** | 0.395** | -0.159 | 1.000 |  |  |  |  |  |  |
| **7th day PDI** | -0.231 | -0.188 | 0.064 | -0.221 | 1.000 |  |  |  |  |  |
| **14th day PDI** | -0.287* | -0.303* | 0.032 | -0.026 | 0.179 | 1.000 |  |  |  |  |
| **21st day PDI** | -0.593** | -0.464** | 0.157 | -0.322* | 0.091 | 0.705** | 1.000 |  |  |  |
| **28th day PDI** | -0.661** | -0.504** | 0.084 | -0.381** | 0.164 | 0.669** | 0.780** | 1.000 |  |  |
| **Average PDI** | -0.612** | -0.493** | 0.109 | -0.314* | 0.194 | 0.835** | 0.920** | 0.935** | 1.000 |  |
| **AUDPC** | -0.564** | -0.467** | 0.114 | -0.269* | 0.182 | 0.874** | 0.946** | 0.869** | 0.988** | 1.000 |

** -significance value at 0.01%, * -significance value at 0.05%
