# Supplementary material for: Deciphering environmental factors and defense response of rice genotypes against sheath blight disease
Source: Physiol Mol Plant Pathol. 2022 Nov;122:101916. doi: 10.1016/j.pmpp.2022.101916 (PMC9669783; doi:10.1016/j.pmpp.2022.101916)
Supplement: Multimedia component 4 [file mmc4.docx]

**Table S4 Correlation among traits associated with sheath blight disease during wet season 2018.**

| **Characters** | **Plant height** | **Panicle length** | **No. of tillers/plant** | **Days to 50% flowering** | **7th day PDI** | **14th day PDI** | **21st day PDI** | **28th day PDI** | **Average PDI** | **AUDPC** |
| --- | --- | --- | --- | --- | --- | --- | --- | --- | --- | --- |
| **Plant height** | 1.000 |  |  |  |  |  |  |  |  |  |
| **Panicle length** | 0.329** | 1.000 |  |  |  |  |  |  |  |  |
| **No. of tillers/plant** | -0.144 | -0.233 | 1.000 |  |  |  |  |  |  |  |
| **Days to 50% flowering** | 0.444** | 0.423** | -0.129 | 1.000 |  |  |  |  |  |  |
| **7th day PDI** | -0.236 | -0.083 | 0.434** | -0.099 | 1.000 |  |  |  |  |  |
| **14th day PDI** | -0.019 | -0.037 | 0.238 | -0.056 | 0.557** | 1.000 |  |  |  |  |
| **21st day PDI** | -0.092 | 0.133 | 0.185 | 0.000 | 0.314* | 0.477** | 1.000 |  |  |  |
| **28th day PDI** | -0.137 | 0.031 | 0.103 | 0.008 | 0.264* | 0.500** | 0.787** | 1.000 |  |  |
| **Average PDI** | -0.123 | 0.044 | 0.226 | -0.022 | 0.492** | 0.745** | 0.891** | 0.908** | 1.000 |  |
| **AUDPC** | -0.103 | 0.052 | 0.250* | -0.030 | 0.528** | 0.800** | 0.895** | 0.834** | 0.988** | 1.000 |

** -significance value at 0.01%, * -significance value at 0.05%
