# Supplementary material for: Deciphering environmental factors and defense response of rice genotypes against sheath blight disease
Source: Physiol Mol Plant Pathol. 2022 Nov;122:101916. doi: 10.1016/j.pmpp.2022.101916 (PMC9669783; doi:10.1016/j.pmpp.2022.101916)
Supplement: Multimedia component 5 [file mmc5.docx]

**Table S5. Population structure group of rice genotypes based on inferred ancestry values.**

| **S. no.** | **Genotypes** | **Inferred ancestry** | | **Structure group** | **Response to sheath blight** |
| --- | --- | --- | --- | --- | --- |
|  |  | **Q1** | **Q2** |  |  |
| 1 | IC277237 | 0.015 | 0.985 | SP2 | MS |
| 2 | IC277317 | 0.989 | 0.011 | SP1 | MS |
| 3 | IC277319 | 0.872 | 0.128 | SP1 | MS |
| 4 | IC278776 | 0.993 | 0.007 | SP1 | MS |
| 5 | IC282454 | 0.939 | 0.061 | SP1 | MS |
| 6 | IC256613 | 0.312 | 0.688 | AD | MS |
| 7 | IC256616 | 0.990 | 0.010 | SP1 | S |
| 8 | IC256617 | 0.965 | 0.035 | SP1 | MS |
| 9 | IC256754 | 0.975 | 0.025 | SP1 | MS |
| 10 | IC256807 | 0.007 | 0.993 | SP2 | MS |
| 11 | IC260917 | 0.991 | 0.009 | SP1 | MR |
| 12 | IC264141 | 0.991 | 0.009 | SP1 | S |
| 13 | IC264151 | 0.010 | 0.990 | SP2 | MS |
| 14 | IC274377 | 0.909 | 0.091 | SP1 | MS |
| 15 | IC274408 | 0.879 | 0.121 | SP1 | MS |
| 16 | IC277248 | 0.988 | 0.012 | SP1 | MS |
| 17 | IC277261 | 0.991 | 0.009 | SP1 | MS |
| 18 | IC277266 | 0.005 | 0.995 | SP2 | MS |
| 19 | IC277274 | 0.499 | 0.501 | AD | MS |
| 20 | IC277284 | 0.971 | 0.029 | SP1 | MS |
| 21 | IC277290 | 0.048 | 0.952 | SP2 | MS |
| 22 | IC277330 | 0.005 | 0.995 | SP2 | MS |
| 23 | IC277332 | 0.451 | 0.549 | AD | MS |
| 24 | IC278777 | 0.011 | 0.989 | SP2 | MS |
| 25 | IC279355 | 0.484 | 0.516 | AD | MS |
| 26 | IC280478 | 0.289 | 0.711 | AD | MS |
| 27 | IC280504 | 0.990 | 0.010 | SP1 | MS |
| 28 | IC280528 | 0.041 | 0.959 | SP2 | MS |
| 29 | IC280564 | 0.990 | 0.010 | SP1 | MS |
| 30 | IC281508 | 0.142 | 0.858 | SP2 | MS |
| 31 | IC281783 | 0.078 | 0.922 | SP2 | MS |
| 32 | IC281786 | 0.005 | 0.995 | SP2 | MS |
| 33 | IC282438 | 0.005 | 0.995 | SP2 | MS |
| 34 | IC282460 | 0.993 | 0.007 | SP1 | MS |
| 35 | IC282463 | 0.006 | 0.994 | SP2 | MS |
| 36 | IC282466 | 0.006 | 0.994 | SP2 | MS |
| 37 | IC282473 | 0.834 | 0.166 | SP1 | MS |
| 38 | IC282480 | 0.008 | 0.992 | SP2 | MS |
| 39 | IC282500 | 0.010 | 0.990 | SP2 | MS |
| 40 | IC282512 | 0.131 | 0.869 | SP2 | S |
| 41 | IC282526 | 0.547 | 0.453 | AD | MS |
| 42 | IC282812 | 0.658 | 0.342 | AD | MR |
| 43 | IC282815 | 0.042 | 0.958 | SP2 | MS |
| 44 | IC283023 | 0.240 | 0.760 | AD | MR |
| 45 | IC283026 | 0.244 | 0.756 | AD | MR |
| 46 | IC283028 | 0.600 | 0.400 | AD | MS |
| 47 | IC283038 | 0.431 | 0.569 | AD | MR |
| 48 | IC283041 | 0.540 | 0.460 | AD | MR |
| 49 | IC283139 | 0.685 | 0.315 | AD | MR |
| 50 | IC283204 | 0.084 | 0.916 | SP2 | MS |
| 51 | IC283207 | 0.988 | 0.012 | SP1 | MS |
| 52 | IC256538 | 0.132 | 0.868 | SP2 | MS |
| 53 | IC277267 | 0.905 | 0.095 | SP1 | MR |
| 54 | IC277275 | 0.988 | 0.012 | SP1 | MS |
| 55 | IC281774 | 0.988 | 0.012 | SP1 | MS |
| 56 | IC283187 | 0.054 | 0.946 | SP2 | MS |
| 57 | IC283206 | 0.363 | 0.637 | AD | MS |
| 58 | CO 39 | 0.975 | 0.025 | SP1 | S |
| 59 | Teqing | 0.817 | 0.183 | SP1 | MS |
| 60 | Jasmine-85 | 0.956 | 0.044 | SP1 | MS |
| 61 | Tapaswini | 0.042 | 0.958 | SP2 | S |
| 62 | Pusa Basmati-1 | 0.891 | 0.109 | SP1 | HS |
| 63 | Tetep | 0.982 | 0.018 | SP1 | MR |
